# Absence of cannabinoid 1 receptor in beta cells protects against high-fat/high-sugar diet-induced beta cell dysfunction and inflammation in murine islets

**DOI:** 10.1007/s00125-018-4576-4

**Published:** 2018-03-01

**Authors:** Isabel González-Mariscal, Rodrigo A. Montoro, Máire E. Doyle, Qing-Rong Liu, Michael Rouse, Jennifer F. O’Connell, Sara Santa-Cruz Calvo, Susan M. Krzysik-Walker, Soumita Ghosh, Olga D. Carlson, Elin Lehrmann, Yongqing Zhang, Kevin G. Becker, Chee W. Chia, Paritosh Ghosh, Josephine M. Egan

**Affiliations:** 1 Laboratory of Clinical Investigation, National Institute on Aging, National Institutes of Health, 251 Bayview Boulevard, Baltimore, MD 21224 USA; 2 Laboratory of Genetics, National Institute on Aging, National Institutes of Health, Baltimore, MD USA; 3 Intramural Research Program, National Institute on Aging, National Institutes of Health, Baltimore, MD USA

**Keywords:** Beta cell, Beta cell proliferation, Beta cell viability, Cannabinoid 1 receptor, Diabetes, Inflammation, Insulin, Insulin resistance, Islet of Langerhans, Mouse

## Abstract

**Aims/hypothesis:**

The cannabinoid 1 receptor (CB1R) regulates insulin sensitivity and glucose metabolism in peripheral tissues. CB1R is expressed on pancreatic beta cells and is coupled to the G protein Gαi, suggesting a negative regulation of endogenous signalling in the beta cell. Deciphering the exact function of CB1R in beta cells has been confounded by the expression of this receptor on multiple tissues involved in regulating metabolism. Thus, in models of global genetic or pharmacological CB1R blockade, it is difficult to distinguish the indirect effects of improved insulin sensitivity in peripheral tissues from the direct effects of inhibiting CB1R in beta cells per se. To assess the direct contribution of beta cell CB1R to metabolism, we designed a mouse model that allows us to determine the role of CB1R specifically in beta cells in the context of whole-body metabolism.

**Methods:**

We generated a beta cell specific *Cnr1* (CB1R) knockout mouse (β-CB1R^−/−^) to study the long-term consequences of CB1R ablation on beta cell function in adult mice. We measured beta cell function, proliferation and viability in these mice in response to a high-fat/high-sugar diet and induction of acute insulin resistance with the insulin receptor antagonist S961.

**Results:**

β-CB1R^−/−^ mice had increased fasting (153 ± 23% increase at 10 weeks of age) and stimulated insulin secretion and increased intra-islet cAMP levels (217 ± 33% increase at 10 weeks of age), resulting in primary hyperinsulinaemia, as well as increased beta cell viability, proliferation and islet area (1.9-fold increase at 10 weeks of age). Hyperinsulinaemia led to insulin resistance, which was aggravated by a high-fat/high-sugar diet and weight gain, although beta cells maintained their insulin secretory capacity in response to glucose. Strikingly, islets from β-CB1R^−/−^ mice were protected from diet-induced inflammation. Mechanistically, we show that this is a consequence of curtailment of oxidative stress and reduced activation of the NLRP3 inflammasome in beta cells.

**Conclusions/interpretation:**

Our data demonstrate CB1R to be a negative regulator of beta cell function and a mediator of islet inflammation under conditions of metabolic stress. Our findings point to beta cell CB1R as a therapeutic target, and broaden its potential to include anti-inflammatory effects in both major forms of diabetes.

**Data availability:**

Microarray data have been deposited at GEO (GSE102027).

**Electronic supplementary material:**

The online version of this article (10.1007/s00125-018-4576-4) contains peer-reviewed but unedited supplementary material, which is available to authorised users.

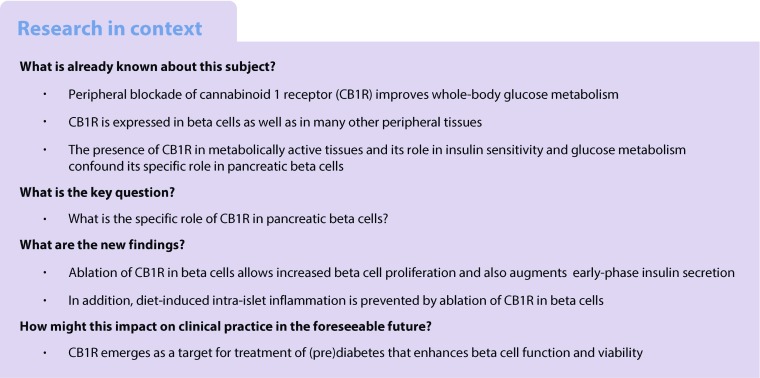



## Introduction

Insulin secretion is tightly controlled as dysregulation has life-threatening consequences. When chronically stimulated, beta cell function deteriorates, and insufficient insulin secretion, especially when coupled with insulin resistance, causes glucose elevation and type 2 diabetes. Glucose metabolism is the primary trigger for insulin secretion, while other factors such as gastric inhibitory polypeptide (GIP) and glucagon-like peptide (GLP-1), cholecystokinin, amino acids (leucine and arginine) and acetylcholine enhance glucose-mediated insulin secretion [1, 2]. Negative regulators exist, with adrenaline (epinephrine), noradrenaline (norepinephrine) and somatostatin being the most studied [1, 3]. The endocannabinoid system (ECS) also has a regulatory role in beta cells. In humans and rodents, beta cells express the cannabinoid 1 receptor (CB1R, encoded by *Cnr1*), synthesise endocannabinoids (ECs) in a glucose-dependent manner [4, 5] and contain the enzymes necessary for EC degradation [4, 6–8]. In obesity, the ECS becomes overactive [8, 9].

CB1R is coupled to the G protein type G_i/o_, Gαi, which inhibits adenylyl cyclase (AC) and cAMP–protein kinase A signalling, activates mitogen-activated protein kinases (MAPKs) and stimulates ceramide synthesis in many tissues [10–13]. It also inhibits voltage-gated L-, N- and P/Q-type Ca^2+^ channels, inwardly rectifying K^+^ channels and leading to inhibition of signal transmission and release of secretory products [14, 15]. These complex but connected signalling pathways impact on many physiological processes, including exocytosis, cell survival and differentiation, metabolism and immune cell responses [11, 16]. Coupling CB1R to the G_αi_ protein indicates a potential negative regulatory role in beta cells, where AC positively regulates insulin secretion and beta cell function. We and others have dissected the actions of CB1R in metabolically active tissues using whole-body *Cnr1* (CB1R) knockout (CB1KO) rodents [17, 18] and pharmacological approaches [8, 19–24]. Nonetheless, the complexity of CB1R’s action, its presence in numerous tissues and the potential off-target effects of pharmacological approaches have confounded the study of CB1R in beta cells. The effects of specifically targeting CB1R in pancreatic beta cells and whether this has direct effects on beta cell function and/or whole-body metabolism have not been studied.

To further interrogate the functions of CB1R in adult beta cells, and to build upon previous work on CB1R blockers as therapeutic agents for obesity-related disorders, we generated an inducible beta cell specific CB1R knockout (β-CB1R^−/−^) mouse and studied the implications of CB1R ablation in beta cells under conditions of acute and chronic insulin resistance in vivo and ex vivo.

## Methods

For detailed methods, please refer to the electronic supplementary material (ESM) Methods.

### Materials

All materials and mouse strains used in this study are detailed in ESM Tables 1–7.

### Animals

Animal care and procedures were approved by the National Institute on Aging Animal Care and Use Committee. Mice were housed in groups of four using 12 h dark/light cycles, provided with water and fed ad libitum. *Cnr1*^flox/flox^ mice were generated as described in the ESM Methods. *Cnr1*^flox/flox^ mice were mated with MIP-Cre/ERT mice (University of Chicago, Chicago, IL, USA) and injected daily for 5 days with i.p. tamoxifen to obtain β-CB1R^−/−^ mice (*Cnr1*^flox/flox^:MIP-Cre/ERT^+^), β-CB1R^+/+^ control littermates (*Cnr1*^flox/flox^:MIP-Cre/ERT^−^) and MIP-Cre/ERT mice (*Cnr1*^wt/wt^:MIP-Cre/ERT^+^). CB1KO mice (NIH, Bethesda, MD, USA) were bred as described in the ESM Methods. Male mice (*n* = 6–7 mice/group) were aged to 25 weeks and body weights and metabolic variables were analysed. Body composition was analysed using NMR. Pancreases were fixed and processed for immunohistochemistry (anti-insulin [1:100; Dako], anti-glucagon [1:500; Sigma-Aldrich], anti-BrdU [1:100; Accurate Chemicals]). Islet size was quantified using Pancreas++ [25]. Hormones were quantified using ELISA. Methanol–chloroform-extracted ECs from plasma were analysed using LC-MS/MS, as described in the ESM Methods.

### Induction of acute insulin resistance

Male mice (*n* = 8–11 mice/group) were randomised to receive vehicle or S961 (0.05 nmol/h) via ALZET miniosmotic pumps. Blood glucose and insulin levels were measured from tail bleed at 0–6 days; mice were injected with BrdU (0.1 nmol/g) and euthanised 12 h later.

### Diet-induced obesity

Male mice (6–8 weeks old; *n* = 6–7/group) were randomised to a standard diet (16.7% kJ fat and 12.4% kJ sugar wt/wt) or high-fat/high-sugar diet (HFHS; 49.2% kJ fat and 32.2% kJ sugar wt/wt) for 15 weeks. Body weight and food consumption were measured weekly. At the end of the study, GTTs and metabolic measurements were performed. Metabolic measurements were measured using a Columbus Instruments Comprehensive Lab Monitoring System (CLAMS; Columbus Instruments), as described in ESM. Tissues (pancreas, liver and white adipose tissue) were dissected, weighed and flash frozen or fixed for immunohistochemistry (anti-CD3, anti-CD68, anti-thioredoxin-interacting protein [TXNIP] and anti-phospho [p][S536]-p65 [1:100; Abcam], anti-ceramide [1:10; Enzo Life Sciences]). Immunoprecipitated insulin receptor (IR) or IRS-2 from liver protein extracts (anti-IRS2 [Cell Signaling], anti-IR [Santa Cruz Biotechnology]; 1 μg) were subjected to Tris-glycine PAGE, immunoblotted with anti-IRS2 or anti-p-Tyr (1:1000; Santa Cruz Biotechnology), visualised by enhanced chemiluminescence and quantified using ImageJ.

### Ex vivo analysis of viability and metabolism of isolated islets

Freshly isolated islets were cultured in non-stimulatory conditions (2 mmol/l glucose) to quantify basal cAMP (*n* = 100 islets/mouse) and resting insulin secretion (*n* = 40 islets/mouse). Islets were cultured with exendin-4 (Ex-4) and 3-isobutyl-1-methylxanthine (IBMX; 25 μmol/l; Sigma-Aldrich), and intra-islet cAMP levels and insulin secretion were quantified. Islets (*n* = 100–120 islets/mouse) were infused with glucose (7.5 mmol/l) in a perifusion system and insulin secretion was quantified. Freshly isolated islets (*n* = 30 islets/group) were used to measure the oxygen consumption rate (OCR), extracellular acidification rate (ECAR) using an XFe24 Seahorse Analyzer (Agilent) and intra-islet levels of reactive oxygen species (ROS) as described in ESM. Islets (*n* = 30–50 islets/group) were cultured in the presence or absence of 500 μmol/l palmitate and 16.5 mmol/l glucose for 24 h. Islet viability, cAMP levels, mRNA expression (*Il1b*, *Nlrp3*, *Tnfa* [also known as *Tnf*], *Ldha*), cytokine secretion (IL-1β, TNF-α), protein phosphorylation (p-Erk1/2, p-SAPK/JNK, p-p38, p-p53, p-Stat1, p-HSP27) and cleavage (caspase 3, PARP) were measured. Islets (*n* = 15 islets/group) were incubated with a mixture of cytokines (10 ng/ml IL-1β, 50 ng/ml TNF-α and 50 ng/ml IFN-γ) or vehicle for 18 h and cytotoxicity was assayed. There were at least three replicates in all experiments.

### Flow cytometry

Pancreatic lymphocytes were isolated after pancreas perfusion and cells were stained using PE anti-mouse CD8a and FITC anti-mouse CD69 (Biolegend), as detailed in the ESM Methods. Populations were determined by using BD FACSCanto II and BD FACSDiva software (BD Biosciences).

### Microarray analysis

Microarray experiments and analysis were performed as previously described [26].

### Method of randomisation

Age and sex-matched littermate mice were randomly assigned to vehicle, S961, standard diet or HFHS.

### Quantification and statistical analysis

Data are presented as means ± SEM. No statistical method was used to predetermine the sample size. Differences between mean values for variables within individual experiments were compared statistically using Student’s *t* test or ANOVA, as appropriate. Comparisons were performed using GraphPad Prism version 6.0. *p* ≤ 0.05 was considered statistically significant.

## Results

### β-CB1R^−/−^ mice have increased beta cell proliferation and improved glucose homeostasis

**Fig. 1:**
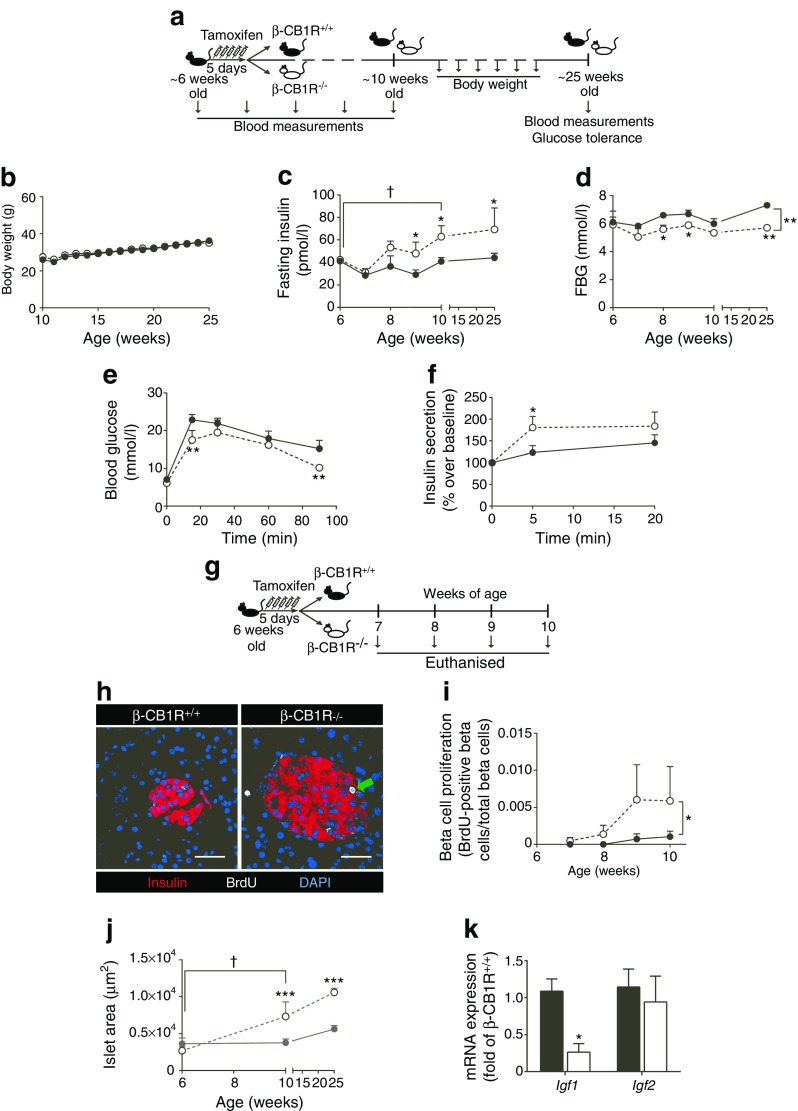
Insulin levels, glucose levels and beta cell proliferation in β-CB1R^−/−^ mice. (**a**) Schematic of the experimental design. Six-week-old MIP-Cre/ERT-*Cnr1*^flox/flox^ mice were injected with i.p. tamoxifen for 5 consecutive days. (**b**) Body weight, (**c**) fasting plasma insulin and (**d**) FBG levels in β-CB1R^+/+^ and β-CB1R^−/−^ mice were measured at the indicated ages. (**e**) IPGTTs and (**f**) mixed glucose–lipid stimulated insulin secretion were examined at 25 weeks of age. (**g**) Mice were injected with BrdU prior to euthanasia at 8, 9 and 10 weeks of age. (**h**) Immunostaining for insulin (red), BrdU (white; green arrow) and nuclei (DAPI; blue) in pancreases from β-CB1R^+/+^ and β-CB1R^−/−^ mice (scale bars, 50 μm). (**i**) Analysis of beta cell proliferation (% BrdU-positive beta cells/total beta cells) and (**j**) islet area (*n* = 100 islets/group). (**k**) Relative mRNA expression of *Igf1* and *Igf2* in isolated islets. *n* = 6 mice in 6- and 25-week old β-CB1R^+/+^ groups and in 6- and 8-week old β-CB1R^−/−^ groups; *n* = 7 mice in 7–10-week old β-CB1R^+/+^ groups and in 7-, 9-, 10- and 25-week old β-CB1R^−/−^ groups. Black circles/bars, β-CB1R^+/+^; white circles/bars, β-CB1R^−/−^. Data are means ± SEM. Significance by ANOVA or *t* test, *p ≤ 0.05, **p ≤ 0.01, ***p ≤ 0.001 vs β-CB1R^+/+^; ^†^*p* ≤ 0.05 vs week 0. See also ESM Figs. 1,2

Conditional β-CB1R^−/−^ mice were obtained by mating *Cnr1*^flox/flox^ with MIP-Cre/ERT mice (ESM Fig. 1). Six-week old β-CB1R^−/−^ and β-CB1R^+/+^ mice were injected with tamoxifen for 5 days and monitored until 25 weeks of age (Fig. 1a–d). No difference in body weight (Fig. 1b) or body composition (ESM Fig. 2a–d) was observed between β-CB1R^+/+^ and β-CB1R^−/−^ mice. Fasting plasma insulin levels were significantly increased (153 ± 23%) by 10 weeks of age in β-CB1R^−/−^ mice compared with baseline and were significantly higher than in β-CB1R^+/+^ (Fig. 1c) and MIP-Cre/ERT mice (ESM Fig. 2e), which was reflected in lower fasting blood glucose (FBG) levels (Fig. 1d, ESM Fig. 2f). β-CB1R^−/−^ mice were more glucose tolerant than β-CB1R^+/+^ mice in an IPGTT (Fig. 1e). After oral glucose-lipid challenge, β-CB1R^−/−^ mice had higher circulating insulin levels than β-CB1R^+/+^ mice (Fig. 1f, ESM Fig. 2g). At 10 weeks, beta cell proliferation was increased 5.6-fold in β-CB1R^−/−^ mice (Fig. 1g–i), and the islet area was significantly greater (1.9-fold) than in β-CB1R^+/+^ (Fig. 1j) and MIP-Cre/ERT (ESM Fig. 2h, i) mice, with no change in islet number (ESM Fig. 2j). Beta cell proliferation is regulated by insulin, cytokines and prolactin, which stimulate *Igf1* expression in beta cells [27]. The trophic changes observed were independent of intra-islet IGF-1, since *Igf1* expression was decreased in β-CB1R^−/−^ compared with β-CB1R^+/+^ islets (Fig. 1k).

### CB1R ablation in beta cells reduced hyperglycaemia during acute insulin resistance

**Fig. 2:**
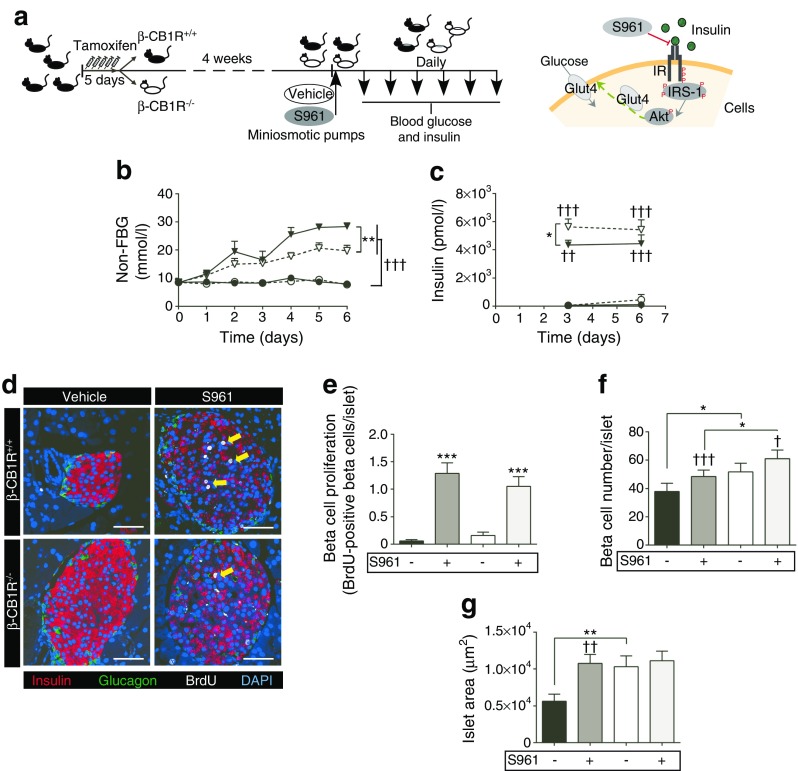
β-CB1R^−/−^ mice are more resistant to the hyperglycaemic effects of S961 than β-CB1R^+/+^ mice. (**a**) Schematic of the experimental design. Miniosmotic pumps containing vehicle or 10 nmol S961 (1.2 nmol/day) were implanted subcutaneously in 10- to 12-week-old β-CB1R^+/+^ and β-CB1R^−/−^ mice 1 month after tamoxifen injections. (**b**) Non-FBG was measured daily and (**c**) plasma insulin levels were measured at days 3 and 6 after pump implantation. (**d**) Immunostaining for insulin (red), glucagon (green), BrdU (white; yellow arrows) and nuclei (DAPI; blue) of pancreases (scale bars, 50 μm). (**e**) Quantification of beta cell proliferation (BrdU-positive beta cells per islet), (**f**) beta cell number per islet and (**g**) islet area. *n* = 100 islets/group. *n* = 8 S961-β-CB1R^+/+^, *n* = 11 S961-β-CB1R^−/−^ mice, *n* = 9 vehicle mice. Black circles/bars, β-CB1R^+/+^ mice treated with vehicle; black triangles/dark grey bars, β-CB1R^+/+^ mice treated with S961; white circles/bars, β-CB1R^−/−^ mice treated with vehicle; white triangles/light grey bars, β-CB1R^−/−^ mice treated with S961. Data are means ± SEM. Significance by *t* test, *p ≤ 0.05, **p ≤ 0.01, ***p ≤ 0.001 vs β-CB1R^+/+^ and ^†^*p* ≤ 0.05, ^††^*p* ≤ 0.01, ^†††^*p* ≤ 0.001 vs vehicle. See also ESM Fig. 3

We examined whether β-CB1R^−/−^ mice would still respond to acute insulin resistance using S961, a competitive antagonist of the IR [28]. Ten-week-old β-CB1R^+/+^ and β-CB1R^−/−^ mice were implanted with miniosmotic pumps containing vehicle or S961. Glucose and insulin levels were followed daily for 6 days (Fig. 2a). By day 6, non-FBG was significantly lower in S961-treated β-CB1R^−/−^ mice (19.7 ± 1.9 mmol/l) than in β-CB1R^+/+^ (28.3 ± 1.3 mmol/l) (Fig. 2b) and MIP-Cre/ERT mice (ESM Fig. 3a). S961 treatment increased circulating insulin levels in β-CB1R^+/+^ mice, but even more so in β-CB1R^−/−^ mice (Fig. 2c). By day 6, S961 had caused a 21-fold increase in beta cell proliferation per islet in β-CB1R^+/+^ mice compared with baseline, and to a lesser extent (6.5-fold) in β-CB1R^−/−^ mice (Fig. 2d, e, ESM Fig. 3b). Increased beta cell proliferation in S961-β-CB1R^+/+^ mice resulted in increased beta cell number and larger islets (Fig. 2f, g). β-CB1R^−/−^ mice already had significantly more beta cells than β-CB1R^+/+^ mice; a 6.5-fold increase in proliferation in β-CB1R^−/−^ mice was sufficient to further increase total beta cell numbers when mice were treated with S961 (Fig. 2f). Alpha cell numbers were comparable between strains; S961 treatment resulted in increased alpha cell numbers only in β-CB1R^+/+^ mice (ESM Fig. 3c). S961 treatment did not lead to a further increase in islet size in β-CB1R^−/−^ mice compared with β-CB1R^+/+^ mice (Fig. 2g) because beta cell turnover was close to its maximum potential due to the absence of CB1R, and a further slight increase in proliferation did not significantly increase size. Beta cell apoptosis, as assessed by TUNEL staining (ESM Fig. 3d, e), and immune cell infiltration were not observed in or around islets (ESM Fig. 3f).

### β-CB1R^−/−^ mice fed an HFHS diet gained more weight and became more glucose intolerant than β-CB1R^+/+^ mice

**Fig. 3:**
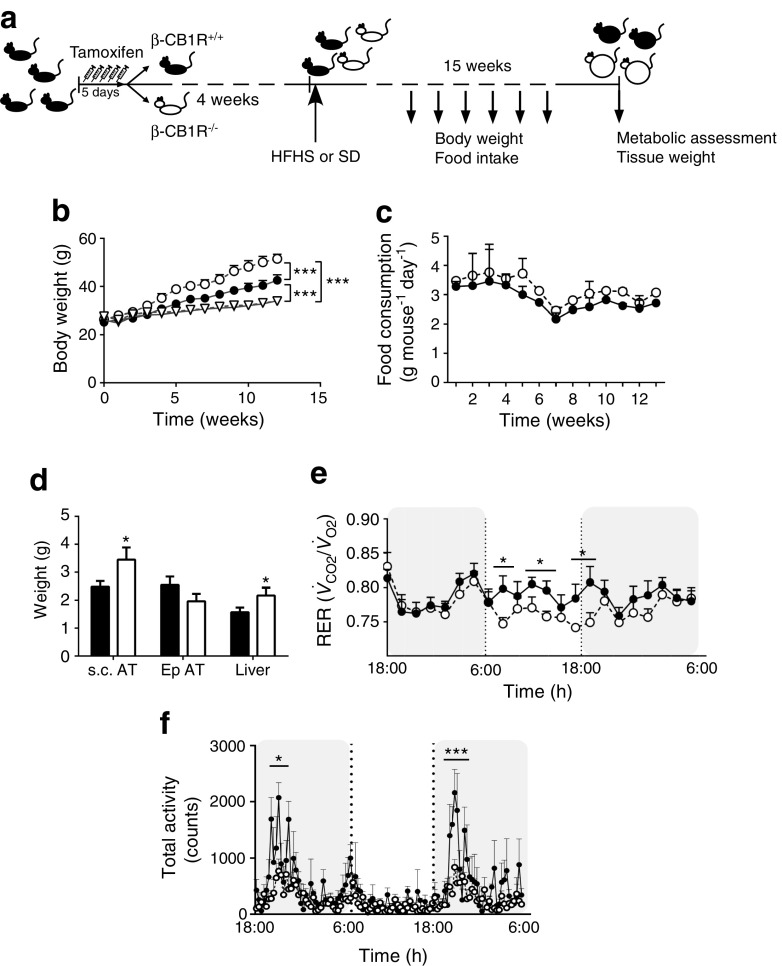
β-CB1R^−/−^ mice become more obese than β-CB1R^+/+^ mice when placed on an HFHS diet. (**a**) Schematic of the experimental design. Six- to 8-week-old mice were injected with tamoxifen and 1 month later were placed on an HFHS diet or standard diet (SD) for 15 weeks. (**b**) Body weight and (**c**) HFHS food intake. (**d**) Liver and epididymal (Ep) and s.c. adipose tissue (AT) were dissected and weighed at week 15. (**e**) Respiratory exchange rate (RER) and (**f**) total physical activity (*x*-axis activity as measured by CLAMS in mice on an HFHS diet. Black triangles, SD-β-CB1R^+/+^ (not visible as covered by the white triangles in **b**); black circles/bars, HFHS-β-CB1R^+/+^; white triangles, SD-β-CB1R^−/−^; white circles/bars, HFHS-β-CB1R^−/−^. *n* = 7 mice/group. Data are means ± SEM. Significance by ANOVA or *t* test, *p ≤ 0.05, ***p ≤ 0.001 vs β-CB1R^+/+^. See also ESM Fig. 4

**Fig. 4:**
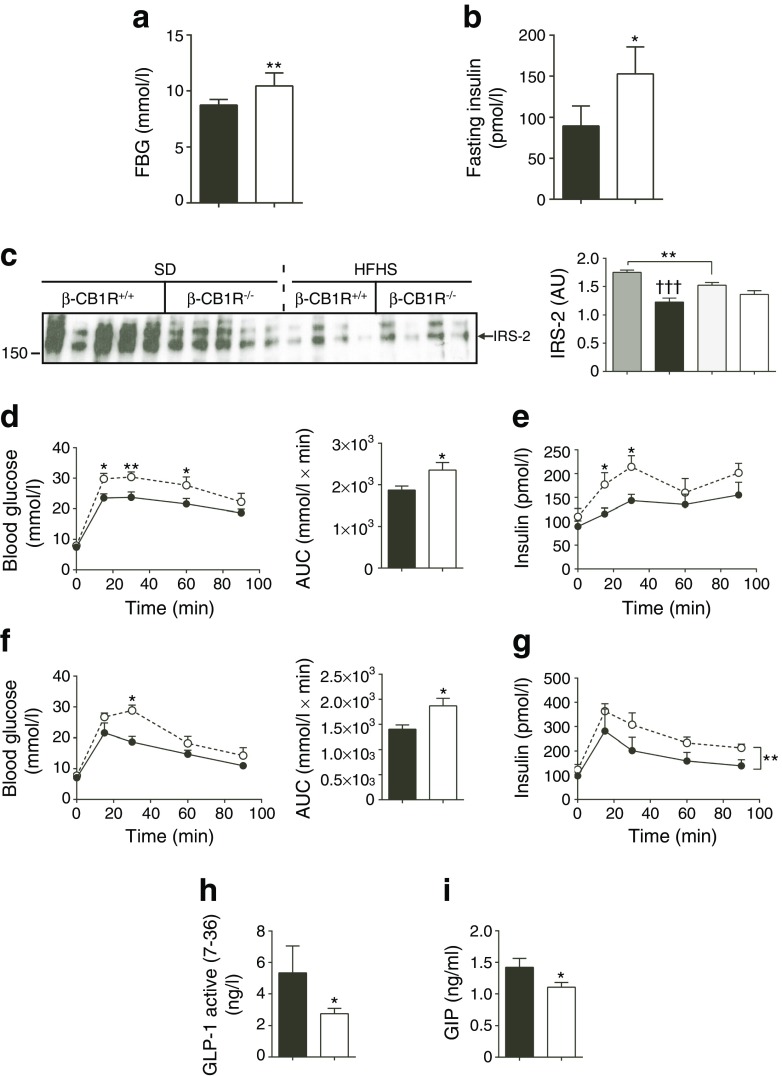
β-CB1R^−/−^ mice become more insulin resistant than β-CB1R^+/+^ mice when placed on an HFHS diet. (**a**) FBG and (**b**) fasting plasma insulin levels. (**c**) Western blot analysis of IRS-2 immunoprecipitated from liver extracts and graph of band density analysed by ImageJ. (**d**, **e**) IPGTT and (**f**, **g**) OGTT (1.5 g/kg glucose) (**d**, **f** blood glucose over time and AUC; **e**, **g** plasma insulin over time). Stimulated (**h**) GLP-1 and (**i**) GIP levels in plasma upon oral glucose–lipid challenge (20 min). *n* = 7 mice. Dark grey bar, SD-β-CB1R^+/+^; black circles/bars, HFHS-β-CB1R^+/+^; light grey bar, SD-β-CB1R^−/−^; white circles/bars, HFHS-β-CB1R^−/−^ . Data are means ± SEM. Significance by ANOVA or *t* test, *p ≤ 0.05, **p ≤ 0.01 vs β-CB1R^+/+^; ^†††^*p* ≤ 0.001 vs standard diet. See also ESM Fig. 5

β-CB1R^+/+^ and β-CB1R^−/−^ mice, 10–12 weeks old, were fed an HFHS diet (HFHS-mice) for 15 weeks (Fig. 3a) as a chronic stimulus for insulin secretion/production. β-CB1R^−/−^ mice had similar body weight (35–36 g) to β-CB1R^+/+^ mice when fed a standard diet (SD-mice). However, HFHS-β-CB1R^−/−^ mice gained 21 ± 4% more weight than HFHS-β-CB1R^+/+^ and MIP-Cre/ERT mice (Fig. 3b, ESM Fig. 4), with comparable food intake (Fig. 3c). This phenotype is distinct from the global CB1KO mouse phenotype, with the latter weighing less than SD- or HFHS-fed β-CB1R^+/+^ and β-CB1R^−/−^ mice (ESM Fig. 5a), demonstrating that this effect is specific to CB1R ablation in beta cells. Liver and subcutaneous adipose tissue from HFHS-β-CB1R^−/−^ mice weighed significantly more than that from control littermates (Fig. 3d), while CB1KO mouse liver and subcutaneous adipose tissue weighed less than that from control littermates (ESM Fig. 5b). HFHS-β-CB1R^+/+^ mice had a higher respiratory exchange rate (RER) during daylight compared with HFHS-β-CB1R^−/−^ mice (0.79 ± 0.01 vs 0.74 ± 0.01) (Fig. 3e). RER, the ratio of carbon dioxide production/oxygen consumption, estimates the fuel source since glucose metabolism requires more oxygen than fat [29]. At night-time the fuel source is the consumed food. The lower RER in HFHS-β-CB1R^−/−^ mice during daylight reflects increased fat utilisation because of differences in fat depots. HFHS-β-CB1R^−/−^ mice had lower activity during the night (active cycle) than HFHS-β-CB1R^+/+^ mice (Fig. 3f). HFHS-β-CB1R^−/−^ mice became more insulin intolerant, as demonstrated by higher FBG (Fig. 4a) and fasting plasma insulin (Fig. 4b) levels than HFHS-β-CB1R^+/+^ mice. Liver IRS-2 protein levels, an indicator of insulin resistance [30], were lower in HFHS-β-CB1R^+/+^ compared with SD-β-CB1R^+/+^ mice (Fig. 4c, ESM Fig. 5c). SD-β-CB1R^−/−^ mice had lower liver IRS-2 levels than SD-β-CB1R^+/+^ mice, which were unchanged on an HFHS diet (Fig. 4c, ESM Fig. 5c). This suggests that even on a standard diet, insulin resistance occurs in the livers of β-CB1R^−/−^ mice, probably as an adaptation to mitigate hypoglycaemia. IR phosphorylation was also decreased in HFHS-β-CB1R^−/−^ mice compared with HFHS-β-CB1R^+/+^ mice (ESM Fig. 5d). Blockade of CB1R in the liver alleviates diet-induced insulin resistance by increasing insulin sensitivity [12]. To determine whether acute pharmacological inhibition of peripheral CB1R (i.e. liver and/or muscle) could alleviate insulin resistance in HFHS-β-CB1R^−/−^ mice, we administered the peripherally restricted CB1R antagonist JD-5037 [19] i.p. 30 min before an ITT. JD-5037 increased insulin sensitivity in HFHS-β-CB1R^+/+^ mice (ESM Fig. 5e), and more so in the insulin-intolerant HFHS-β-CB1R^−/−^ mice (ESM Fig. 5f). During an IPGTT, HFHS-β-CB1R^−/−^ mice had higher blood glucose than HFHS-β-CB1R^+/+^ mice, probably because of increased insulin resistance (Fig. 4d). Interestingly, early insulin secretion was protected in HFHS-β-CB1R^−/−^ mice (Fig. 4e). Similar results were obtained during an OGTT (Fig. 4f, g). Stimulated GLP-1 and GIP plasma levels were significantly lower in HFHS-β-CB1R^−/−^ compared with HFHS-β-CB1R^+/+^ mice 20 min after glucose-lipid challenge (Fig. 4h, i), which probably indicates a homeostatic negative feedback response to prevent hyperinsulinaemia.

### CB1R ablation in beta cells leads to enhanced function in isolated islets

**Fig. 5:**
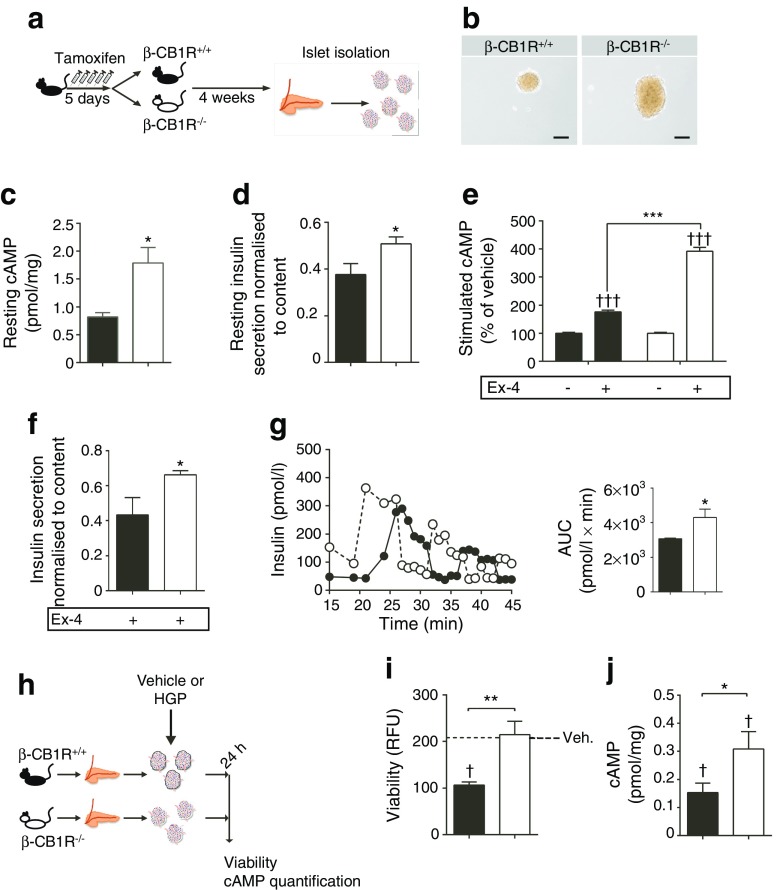
Pancreatic islets from β-CB1R^−/−^ mice secrete more insulin and are protected from HGP stress ex vivo. (**a**) Schematic of the experimental design. Pancreatic islets were isolated from β-CB1R^+/+^ and β-CB1R^−/−^ mice 1 month after tamoxifen injections. (**b**) Representative images of islets (scale bars, 100 μm). (**c**) Resting intra-islet cAMP and (**d**) insulin secretion were quantified under non-stimulatory conditions (2 mmol/l glucose). (**e**) Islets (*n* = 40) were stimulated in vitro with 0.33 nmol/l Ex-4 in the presence of IBMX and 7.5 mmol glucose, and intra-islet cAMP was quantified. (**f**) Ex-4-mediated glucose-dependent insulin secretion from freshly isolated islets. (**g**) Glucose-stimulated insulin secretion from perifused β-CB1R^+/+^ and β-CB1R^−/−^ islets with AUC (*n* = 100 islets; *n* = 4 mice). (**h**) Schematic of the experimental design. Islets isolated from β-CB1R^+/+^ and β-CB1R^−/−^ mice were incubated with HGP (glucose 16.5 mmol/l, palmitate 500 μmol/l) for 24 h. (**i**) Viability of HGP-treated islets (the dotted line represents the vehicle control [Veh.]). RFU, relative fluorescence units. (**j**) Intra-islet cAMP levels after treatment with HGP. Black circles/bars, β-CB1R^+/+^; white circles/bars, β-CB1R^−/−^. *n* = 7 mice; *n* = 3 independent experiments with *n* = 40–50 islets. Data are means ± SEM. Significance by *t* test, *p ≤ 0.05, **p ≤ 0.01, ***p ≤ 0.001 vs β-CB1R^+/+^; ^†^*p* ≤ 0.05, ^†††^*p* ≤ 0.001 vs vehicle

To study the enhanced beta cell function observed in β-CB1R^−/−^ mice, we isolated islets for ex vivo analysis (Fig. 5a). β-CB1R^−/−^ islets were visually bigger than those from β-CB1R^+/+^ mice (Fig. 5b), consistent with in situ measurements (Fig. 1j). CB1R is constitutively active under non-stimulated conditions, and thus inverse agonists or genetic ablation should eliminate basal activity [31]. Non-stimulated, resting intracellular cAMP levels (Fig. 5c) and insulin secretion (Fig. 5d) were increased in β-CB1R^−/−^ compared with β-CB1R^+/+^ islets (217 ± 33% and 175 ± 10%, respectively). Ex-4-stimulated AC activity resulted in a 391 ± 9% increase in cAMP levels in β-CB1R^−/−^ islets compared with 176 ± 4% in β-CB1R^+/+^ islets (Fig. 5e). Ex-4-stimulated glucose-mediated insulin secretion was also significantly higher in β-CB1R^−/−^ compared with control islets (Fig. 5f). Islets were placed in a perifusion system [8] to examine glucose-stimulated insulin secretion (Fig. 5g). β-CB1R^−/−^ islets had increased insulin secretion, an earlier peak of the first phase and greater AUC for insulin than β-CB1R^+/+^ islets (Fig. 5g), all likely resulting from increased AC activity (Fig. 5c). Similar results were found in CB1KO islets (ESM Fig. 6a). To mimic diet-induced beta cell damage, we cultured isolated islets with or without high glucose and palmitate (HGP) for 24 h (Fig. 5h). Loss of viability as seen in HGP-β-CB1R^+/+^ islets was not observed in HGP-β-CB1R^−/−^ islets (Fig. 5i). HGP-β-CB1R^−/−^ islets also had higher intracellular cAMP levels (required for maximal insulin secretion) than control littermates, reflecting increased beta cell functional capacity (Fig. 5j).

### CB1R ablation in beta cells leads to a metabolic shift and reduces ROS production

**Fig. 6:**
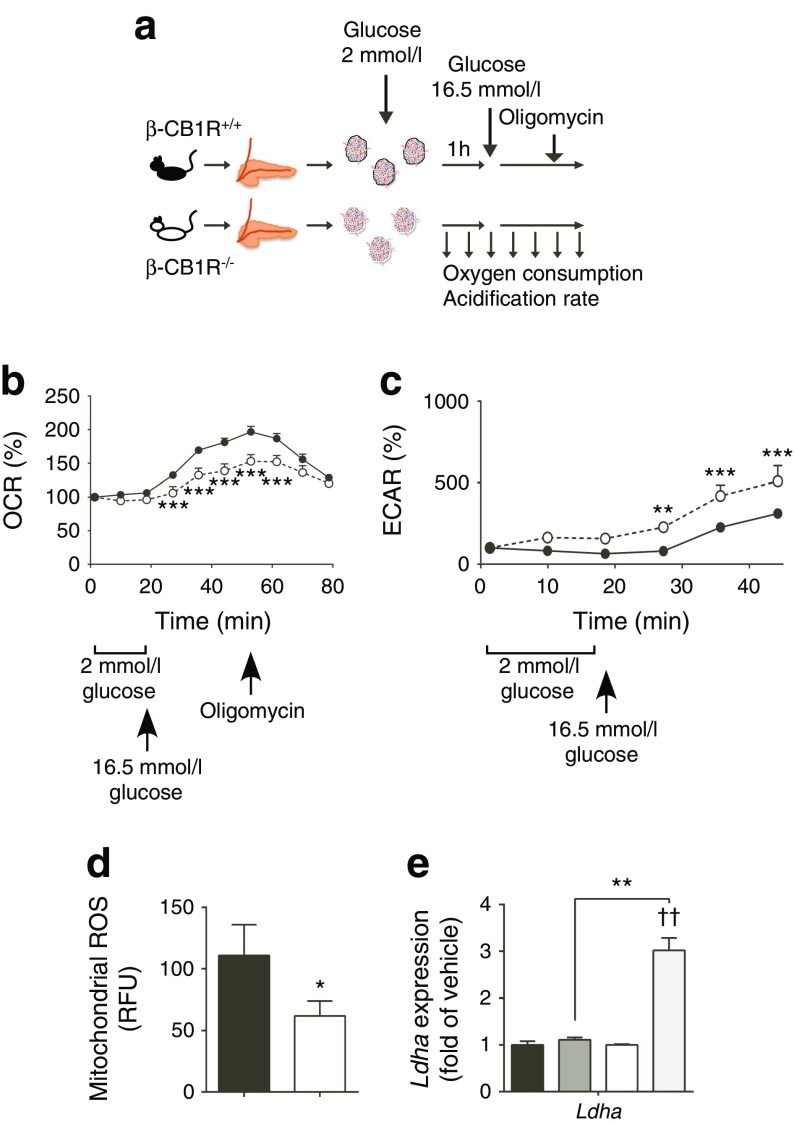
β-CB1R^−/−^ islets have a metabolic shift and produce fewer ROS. (**a**) Schematic of the experimental design. Pancreatic islets were isolated from β-CB1R^+/+^ and β-CB1R^−/−^ mice a month after tamoxifen injections. Freshly isolated islets (*n* = 30) were incubated in 2 mmol/l glucose and (**b**) the OCR and (**c**) ECAR were measured using Seahorse Technology when stimulated with 16.5 mmol/l glucose. *n* = 30 islets, *n* = 6–8 replicates. Black circles, β-CB1R^+/+^; white circles, β-CB1R^−/−^. (**d**) Mitochondrial production of ROS in freshly isolated islets. RFU, relative fluorescence units. (**e**) Relative mRNA expression of *Ldha* in HGP-treated β-CB1R^+/+^ (black bar, vehicle; dark grey bar, HGP) and β-CB1R^−/−^ (white bar, vehicle; light grey bar, HGP) islets. *n* = 3 independent experiments with *n* = 30–50 islets. Data are means ± SEM. Significance by *t* test, *p ≤ 0.05, **p ≤ 0.01, ***p ≤ 0.001 vs β-CB1R^+/+^; ^††^*p* ≤ 0.01 vs vehicle. See also ESM Fig. 6

CB1R impacts mitochondrial metabolism in muscle and neurons [32, 33], and ATP generation via glucose metabolism is required for insulin secretion [34]. We analysed mitochondrial metabolism in β-CB1R^−/−^ islets by measuring the OCR and ECAR (Fig. 6a–c). Islets were pre-incubated for 1 h in 2 mmol/l glucose; the addition of 16.5 mmol/l glucose increased mitochondrial respiration (oligomycin-inhibited ΔOCR) by 185 ± 8% in β-CB1R^+/+^ islets, and to a lesser extent (158 ± 9%) in β-CB1R^−/−^ islets (Fig. 6b, ESM Fig. 6b). The OCR was inhibited by oligomycin in both strains, indicating that the oxygen consumption was due to mitochondrial respiration. The basal ECAR (an indicator of lactate production) was equal in both strains, and the addition of glucose significantly increased the ECAR in β-CB1R^−/−^ islets compared with β-CB1R^+/+^ islets (509 ± 94% vs 311 ± 28%; Fig. 6c). Mitochondria are the primary source of ROS, and a high respiration rate is associated with ROS production [35]. Islets from fasted β-CB1R^−/−^ mice had significantly lower ROS production compared with β-CB1R^+/+^ mice (Fig. 6d). *Ldha* expression significantly increased only in HGP-β-CB1R^−/−^ islets (Fig. 6e), in concordance with the ECAR data.

### CB1R ablation in beta cells protects islets from metabolic stress and inflammation

**Fig. 7:**
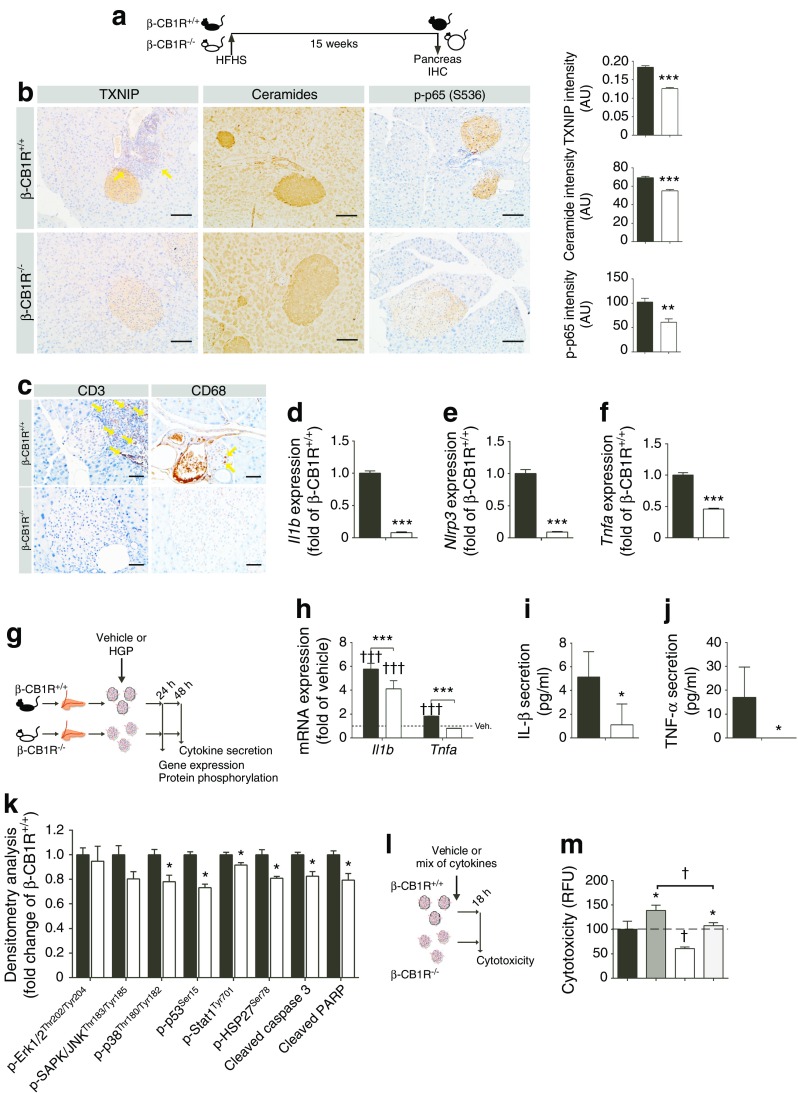
Ablation of CB1R protects islets from HFHS diet-induced inflammation. (**a**) Schematic of the experimental design. β-CB1R^+/+^ and β-CB1R^−/−^ mice were fed an HFHS diet for 15 weeks, after which the mice were euthanised and pancreases were dissected and processed for immunohistochemistry (IHC) and stained. (**b**) Immunostaining for TXNIP, ceramides and p-p65 of pancreases from β-CB1R^+/+^ and β-CB1R^−/−^ mice fed an HFHS diet for 15 weeks (scale bars, 100 μm) and their quantification. (**c**) Immunostaining for CD3 and CD68 in pancreases from β-CB1R^+/+^ and β-CB1R^−/−^ mice fed an HFHS diet (scale bars, 50 μm). The yellow arrows point to areas of immune cell infiltrate between a blood vessel and islet, and into the islet. (**d**–**f**) Relative mRNA expression of inflammatory markers in freshly isolated islets. (**g**) Schematic of the experimental design. Islets isolated from β-CB1R^+/+^ and β-CB1R^−/−^ mice were treated with HGP (glucose 16.5 mmol/l, palmitate 500 μmol/l) or vehicle. (**h**) Relative mRNA expression of inflammatory markers (the dotted line represents the vehicle control). (**i**, **j**) Secretion of inflammatory markers. (**k**) Densitometry of phosphorylated proteins and cleaved proteins from isolated islets treated with HGP. *n* = 7 mice; *n* = 3 independent experiments with *n* = 30–50 islets. Black bars, β-CB1R^+/+^; white bars, β-CB1R^−/−^. (**l**) Schematic of the experimental design. Islets isolated from β-CB1R^+/+^ and β-CB1R^−/−^ mice were treated with vehicle or a mixture of cytokines for 18 h. (**m**) Cytotoxicity of vehicle- or cytokine-treated islets. RFU, relative fluorescence units. Black, β-CB1R^+/+^ mice treated with vehicle; dark grey, β-CB1R^+/+^ mice treated with cytokines; white, β-CB1R^−/−^ mice treated with vehicle; light grey, β-CB1R^−/−^ mice treated with cytokines. *n* = 4 mice; *n* = 4 replicates; *n* = 15 islets. Data are means ± SEM. Significance by *t* test or ANOVA, *p ≤ 0.05, **p ≤ 0.01, ***p ≤ 0.001 vs β-CB1R^+/+^; ^†^*p* ≤ 0.05, ^†††^*p* ≤ 0.001 vs vehicle. See also ESM Fig. 7

Obesity-related molecules such as ROS, ceramides, palmitate and elevated circulating glucose activate the NLRP3 (NLR family, pyrin domain containing 3) inflammasome through phosphorylation of NFκB p65, leading to immune cell infiltration [36–41]. ROS activates NLRP3 by inducing a direct interaction with TXNIP [36–38]. HFHS-β-CB1R^−/−^ mice (Fig. 7a) had lower intra-islet levels of TXNIP and ceramide than HFHS-β-CB1R^+/+^ mice (Fig. 7b). Intra-islet levels of p-p65 were significantly higher in HFHS-β-CB1R^+/+^ compared with HFHS-β-CB1R^−/−^ mice (Fig. 7b). Infiltrating cells, such as T cells (CD3^+^) and macrophages (CD68^+^), were observed in close proximity to blood vessels, and in and around the islets of HFHS-β-CB1R^+/+^ mice (yellow arrows, Fig. 7b, c, ESM Fig. 7a). Flow cytometry analysis showed that the T cells were CD69^+^ (ESM Fig. 7a). Such infiltration was absent in β-CB1R^−/−^ mice fed an HFHS diet for 15 weeks (Fig. 7a, c, ESM Fig. 7b). Circulating EC levels remained unchanged (ESM Fig. 7c) and intra-islet *Cnr2* expression was significantly lower (ESM Fig. 7d) in β-CB1R^−/−^ mice; there was no compensation by the cannabinoid 2 receptor (CB2R), the main EC receptor in immune cells, or activation of CB1R in other islet cell types that explained the reduced immune cell infiltration. β-CB1R^−/−^ islets had lower *Il1b*, *Nlrp3* and *Tnfa* expression than β-CB1R^+/+^ islets (Fig. 7d–f). Microarray analysis confirmed a downregulation of CB1R, TXNIP, NFκB, TNF-α and IL-1β pathways in β-CB1R^−/−^ relative to β-CB1R^+/+^ islets (ESM Fig. 7e). *Tnfa* expression was increased only in HGP-β-CB1R^+/+^ but not in HGP-β-CB1R^−/−^ islets (Fig. 7g, h). Although *Il1b* expression was increased in HGP-β-CB1R^−/−^ islets (Fig. 7h), it was significantly less than in HGP-β-CB1R^+/+^ mice. Secretion of IL-1β and TNF-α from HGP-β-CB1R^−/−^ was significantly reduced or undetectable, respectively, compared with HGP-β-CB1R^+/+^ islets (Fig. 7i, j). Activation of JNK and p38, as happens with proinflammatory cytokines such as IL-1β and TNF-α, leads to beta cell death [42, 43] and EC-mediated cytokine secretion from macrophages [18]. HGP-β-CB1R^−/−^ islets had significantly lower levels of p-p38 than β-CB1R^+/+^ islets, while p-Erk1/2 or p-SAPK/JNK did not change (Fig. 7k). Downstream signalling of p38 was also reduced (p-p53, p-Stat1 and p-HSP27), together with reduced cleaved-caspase 3 and cleaved-PARP (Fig. 7k), indicating reduced cytokine-induced apoptosis [44]. To investigate if lower MAPK activation was due to lower cytokine secretion or sensitivity, islets were treated ex vivo with mixtures of cytokines (IL-1β, TNF-α and IFN- γ [45]) for 18 h (Fig. 7l). β-CB1R^−/−^ islets had significantly lower levels of cytotoxicity than β-CB1R^+/+^ islets, with levels never reaching above those of vehicle-treated β-CB1R^+/+^ islets (Fig. 7m). These data show that β-CB1R^−/−^ islets have lower basal cytotoxicity and, while responsive to cytokines, lower cytokine secretion lessens the inflammatory cascade protecting the islet.

## Discussion

Our study describes the role of CB1R specifically in pancreatic beta cells and its relevance to whole-animal physiology. By genetically ablating CB1R in adult beta cells, we conclude that CB1R is a negative regulator of beta cell function, proliferation and viability, and is a key component of the inflammatory response of islets. ECs orchestrate the organisation of pancreatic islets in perinatal development [46]. Ablating CB1R in adult mice avoids potential alterations in islet structure. Previous reports related to the ECS in beta cells have been conflicting due to the influence of CB1R in other tissues involved in glucose homeostasis and off-target effects of pharmacological approaches; other non-CB1R cannabinoid receptors have been described in islets [5, 47]. While whole-body genetic or pharmacological CB1R blockade has been reported to result in increased insulin secretion [4, 24], enhanced beta cell function may be a consequence of reduced insulin resistance in liver and muscle [10, 11], producing a favourable environment for beta cells. Our model definitively describes the importance of CB1R in beta cell biology and function by eliminating these confounding factors and, for the first time, describes the direct effects of CB1R on beta cell mitochondria. CB1R ablation in beta cells resulted in a metabolic shift towards reduced mitochondrial respiration that protected islets from oxidative stress and improved beta cell functional capacity. Unexpectedly, ablation of CB1R in beta cells was sufficient to prevent the inflammatory response in islets under the metabolic stress of an HFHS diet.

Ablation of CB1R, a constitutively active G_αi_ protein-coupled receptor and AC inhibitor, resulted in increased intra-beta cell cAMP and earlier onset of in vivo and ex vivo insulin secretion. We propose that increased insulin secretion led to increased levels of circulating insulin in β-CB1R^−/−^ mice, eventually resulting in insulin resistance. Isolated β-CB1R^−/−^ islets confirmed increased insulin secretion as a primary event, and our mouse model provides insight into the response of the whole body to primary hyperinsulinaemia. Excessive unremitting insulin secretion due to a primary beta cell event, such as overexpression of the *Ins* gene or chronic exogenous administration of insulin, results in insulin resistance in the liver, weight gain, increased adiposity, ectopic adipose deposition and loss of the ability to increase glucose-stimulated insulin secretion [48, 49]. Primary hyperinsulinaemia with normal blood glucose levels occurs with inactivating mutations of the SUR1 subunit of the K^+^-ATP channel in mice; however, these mice lose their glucose-stimulated insulin secretion capacity time [50]. Therefore, insulin resistance in HFHS-β-CB1R^−/−^ mice is probably a homeostatic response to prevent hypoglycaemia. However, unlike in previous genetic models of primary hyperinsulinaemia, beta cell dysfunction was not observed in HFHS-β-CB1R^−/−^ mice. This is due to a myriad of beneficial intracellular events, an increased beta cell number and a reduced intracellular inflammatory environment. These data underline the potential of CB1R blockade as a treatment for type 2 diabetes compared with classic treatments that elevate insulin secretion, such as sulfonylureas, which do not integrate other signals in beta cells.

An HFHS diet led to greater weight gain in β-CB1R^−/−^ mice. SD- or HFHS-CB1KO mice do not gain weight due to reduced food intake because of CB1R ablation in the brain [51, 52]. Chronic hyperinsulinaemia per se may drive fat deposition in HFHS-β-CB1R^−/−^ mice as it stimulates insulin signalling in white adipose tissue and increases body weight [49, 53, 54]. This is substantiated by our observation that HFHS-β-CB1R^−/−^ mice had a lower RER and decreased activity relative to HFHS-β-CB1R^+/+^ mice, resulting in less glucose uptake by muscle and increased fat deposition. A lower RER in HFHS-β-CB1R^−/−^ mice during daylight probably indicates that the two strains are not using the same stored fuel source. HFHS-β-CB1R^−/−^ mice have more subcutaneous adipose tissue, which may alter the ‘accessibility’ to fuel in the resting state compared with HFHS-β-CB1R^+/+^ mice.

Recently, CB1R was found in the mitochondrial membranes of neurons and muscle [32, 33], but its role in mitochondria has not been described in beta cells. Some reports have indicated that CB1R activation decreases mitochondrial respiration [55] while others have reported the opposite [32]. We found a reduction in mitochondrial respiration in β-CB1R^−/−^ islets and increased *Ldha* expression under the metabolically stressful condition of HGP. Decreased mitochondrial respiration possibly results from nullifying CB1R in beta cell plasma membranes. This could be the result of two effects: increased intracellular cAMP that induces *Ldha* expression [56] and/or increased efficiency of insulin secretion that requires less mitochondrial activation. Regardless, the reduction in mitochondrial respiration led to reduced ROS, thereby probably preventing oxidative stress damage.

**Fig. 8:**
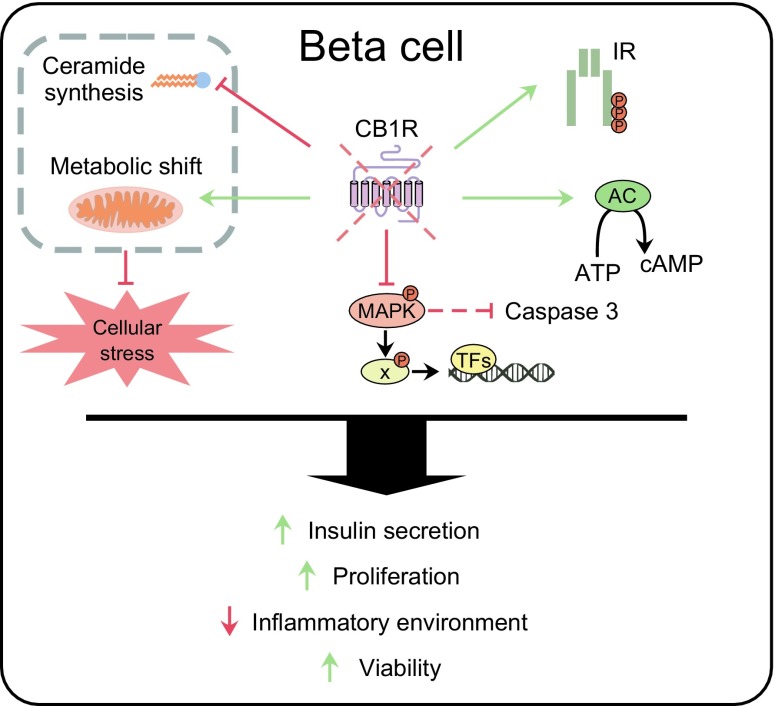
Suggested mechanism of action of CB1R in pancreatic beta cells. Absence of CB1R, or its pharmacological blockade, increases IR sensitivity and AC activity. Ablation of CB1R also blocks ceramide synthesis and induces a metabolic shift towards reduced mitochondrial respiration, reducing cellular oxidative stress. In addition, ablation of CB1R lowers MAPK activation by phosphorylation and therefore its downstream signalling (X), leading to reduced activity of transcription factors (TFs) that regulate inflammatory cytokines and caspase 3 cleavage. Taken together, these changes lead to improved beta cell functional capacity, as demonstrated by increased insulin secretion, increased preservation of beta cell mass by inducing beta cell proliferation, and improved beta cell viability through a reduction in the inflammatory environment

Ablating CB1R in beta cells of adult mice unexpectedly reduced the inflammatory environment in islets. While CB2R is a known immune modulator, it was recently reported that CB1R plays a role in the inflammatory process in the pancreas of a model of type 2 diabetes. Inhibiting *Cnr1* expression using small interfering RNA specifically in macrophages or comprehensive peripheral CB1R blockade by JD-5037 led to reduced activation of the NLRP3 inflammasome in macrophages, and reduced immune infiltration in leptin receptor-deficient Zucker diabetic fatty rat islets [22]. The authors did not implicate CB1R in beta cells as a factor in macrophage-mediated beta cell failure. We now know that CB1R in beta cells is necessary for HFHS-induced inflammation in and around mouse islets. The differing results could be attributed to CB1R ablation in different tissues (macrophages vs beta cells), different stressors (rats fed a standard diet vs mice fed an HFHS diet to more closely mimic the Western diet) and normally functioning leptin receptors present in β-CB1R^−/−^ mice. β-CB1R^−/−^ islets had significantly lower basal expression and lower secretion of proinflammatory cytokines than β-CB1R^+/+^ islets, and reduced activation of p38 compared with β-CB1R^+/+^ islets, which, similar to NFκB, is downstream of IL-1β signalling. A reduction in MAPK activation would further reduce caspase cleavage and activation of proinflammatory transcription factors (Fig. 8). The NLRP3 inflammasome is inhibited by cAMP, and activated by ROS and ceramides [22, 36]. The absence of CB1R facilitated increased cAMP levels and reduced ceramide production, key factors in reducing inflammation in β-CB1R^−/−^ mice. The expression of *Cnr2* was also reduced in β-CB1R^−/−^ islets, most likely due to reduced macrophage infiltration or numbers of resident islet macrophages, since macrophages have high expression levels of *Cnr2*. Moreover, chronic activation of CB1R and CB2R by synthetic agonists in human islets ex vivo did not affect beta cell viability, suggesting that crosstalk between the ECS of beta cells and immune cells is required for HFHS diet-induced immune cell infiltration in and around islets. It has been stated that production of ROS by mitochondria is obligatory for insulin secretion since it is a necessary by-product of ATP production [57]. However excess ROS production, when insulin secretion is under constant high demand such as with an HFHS diet, would eventually be detrimental to beta cells. Lack of CB1R induced a metabolic shift, with a reduction in mitochondrial glucose-stimulated oxygen consumption. ROS production and overall oxidative damage were lowered, aiding in preserving beta cell viability. We observed lower levels of TXNIP, a direct activator of NLRP3 and indicator of ROS damage [36–38], in the islets of HFHS-β-CB1R^−/−^ compared with HFHS-β-CB1R^+/+^ mice.

In summary, CB1R exerts control over beta cell function, proliferation, viability and intracellular signalling (Fig. 8). We can now speculate why an autonomous ECS exists in beta cells. Many toxins that are no longer a threat to humans in the developed world because of vaccinations and antibiotics increase intra-beta cell cAMP, leading to possible death from hypoglycaemia because of hyperinsulinaemia. The presence of a robust intrinsic system, such as the ECS, to reduce intracellular cAMP and limit insulin secretion under duress probably provided a survival advantage. Simultaneously enhancing the ability of beta cells to attract immune cells to eliminate bacterial antigens, cell debris, islet amyloid polypeptide deposition, toxins and plasmids would also have been beneficial. We postulate that the ECS evolved in islets to serve a dual function: to diminish the effects of AC activators while increasing the ability of beta cells to protect themselves. Islets express all the machinery to synthesise ECs on demand [4, 5] and, similar to insulin, ECs (mainly 2-arachidonoylglycerol) are secreted from the islets upon glucose stimulation [4]. Unfortunately our Westernised diets, high in fats and glucose, lead to a near-constant demand for insulin, increased synthesis of islet amyloid polypeptide and possibly increases in bacterial antigens from the gut, increased overall chemokine production, intra-islet inflammation, islet EC secretion, and over-activation of the islet ECS, eventually leading to beta cell dysfunction and hyperglycaemia. Pharmacological blockade of CB1R or hepatocyte CB1R nullification improves insulin action and reduces fat deposition in the liver [12, 19]. Furthermore, CB1R inhibition in the periphery lowers insulin resistance [12, 19]. We now report that CB1R blockade improves beta cell function and protects against HFHS-induced islet inflammation, and may represent a therapeutic strategy in diabetes and impaired glucose tolerance. It has recently been reported that peripheral CB1R blockade in mice with impaired glucose tolerance improved glucose handling and resulted in several anti-inflammatory and cytoprotective actions [58]. Nonetheless our study has the limitation that the rodent and human islet ECS are not identical, and further studies in humans are therefore needed. Previously [8], we found that human beta cells and hepatocytes, but not brain, predominantly express an isoform of CB1R, CB1b. Pharmacological blockade or morpholino oligos [59] to block CB1b mRNA transcription may lead to novel therapies for type 2 diabetes.

## Electronic supplementary material

ESM (PDF 1.77 mb)

## Data Availability

Microarray data have been deposited at GEO (GSE102027). The data generated during the current study are available from the corresponding author on reasonable request.

## Abbreviations

AC: Adenylyl cyclase
CB1KO: Cannabinoid 1 receptor knockout
CB1R: Cannabinoid 1 receptor
β-CB1R^−/−^: Beta cell specific cannabinoid 1 receptor knockout
CB2R: Cannabinoid 2 receptor
CLAMS: Comprehensive Lab Monitoring System
EC: Endocannabinoid
ECAR: Extracellular acidification rate
ECS: Endocannabinoid system
Ex-4: Exendin-4
FBG: Fasting blood glucose
GIP: Gastric inhibitory polypeptide
GLP-1: Glucagon-like peptide
HFHS: High-fat/high-sugar diet
HGP: High glucose and palmitate
IBMX: 3-Isobutyl-1-methylxanthine
IR: Insulin receptor
MAPK: Mitogen-activated protein kinase
NLRP3: NLR family, pyrin domain containing 3
OCR: Oxygen consumption rate
RER: Respiratory exchange rate
ROS: Reactive oxygen species
SD: Standard diet (assignment group)
TXNIP: Thioredoxin-interacting protein
