# Supplementary material for: Absence of cannabinoid 1 receptor in beta cells protects against high-fat/high-sugar diet-induced beta cell dysfunction and inflammation in murine islets
Source: Diabetologia. 2018 Mar 1;61(6):1470–83. doi: 10.1007/s00125-018-4576-4 (PMC6201315; doi:10.1007/s00125-018-4576-4)
Supplement: Supplementary file 1 — (PDF 1.77 mb) [file 125_2018_4576_MOESM1_ESM.pdf]

ESM METHODS

Generation of $Cnr1^{loxP/loxP}$ and β-CB1R$^{-/-}$ mice

Heterozygous *Cnr1*$^{loxP/wt}$ (CB1R gene, i.e. *Cnr1*, flanked by LoxP sites) mice were generated by inGenious Targeting Laboratory (Ronkonkoma, NY, USA). The vector construct was designed inserting a *loxP* cassette 5' 242 bp upstream of the mouse *Cnr1* coding exon, upstream of its starting codon and splicing acceptor site, and a 3' *loxP*-FRT-Neomycin-FRT (Neo) cassette 1,876 bp downstream of the stop codon, in the 3'-UTR sequence of the gene. The vector contained 3.6 kb of gDNA representing the complete coding sequence of the murine *Cnr1* gene and 2.93 kb and 6.3 kb of gDNA as 5' and 3' homologous arms respectively. Cre recombination deleted region includes 242 bp intron sequence with splicing acceptor site, 63 bp of 5'-UTR, translation initiation codon, entire coding region of 1,419 bp, stop codon and 1,876 bp 3'-UTR with partial 1,916 bp 3'-UTR remaining in the KO mice. Ten µg of linearized vector were transfected by electroporation of C57BL/6 X129/SvEv hybrid mouse embryonic stem cells to obtain the floxed-Neo allele. Neomycin-resistant clones were screened for homologous recombination by PCR of gDNA using Lox1/SDL2 primers (427 and 487 bp products for the wild-type and positive *loxP* site respectively). Secondary confirmation of positive clones was performed by Southern Blotting. Screening of internal short arm (5′ end homologous recombination) was achieved using KpnI and probe PB5' with an expected band of 11.75 kb, and screening of external long arm (3′ end homologous recombination) was achieved using MfeI as restriction enzyme and probe PB3' with an expected band is 16.6 kb. Hybrid embryonic cells were microinjected into C57BL/6 blastocysts. Chimeras (F0) were bred to wild-type mice to generate mice bearing the recombinant *Cnr1* allele (F1). F1 mice were bred to C57BL/6 FLP transgenic mice (a mouse ubiquitously expressing FLP recombinase) to remove the FRT-flanked Neo cassette (F2). Neo deletion was confirmed by PCR using NDEL1/ NDEL2 primer set: 255 bp/367 bp for wild-type/Neo-deletion. F2 mice were bred to wild-type mice, and offspring mice lacking FLP recombinase (F3) were selected. F3 mice were back-crossed to C57BL/6 mice until at least the 6th generation. *Cnr1*$^{loxP/wt}$ mice were bred to obtain homozygous, determined by PCR with NDL1/NDL2 primers: expected bands are 367bp (*Cnr1*$^{loxP/loxP}$) 255 and 367 bp (*Cnr1*$^{loxP/wt}$) and 255bp (*Cnr1*$^{wt/wt}$). *Cnr1*$^{loxP/loxP}$ mice were mated to MIP-Cre/ERT mice, a mouse strain expressing Cre-ERT (estrogen receptor-driven Cre) under control of mouse Ins1 promoter, which is specific to beta-cells, with no leakage of Cre in brain (Figure S1D,E), obtaining Cre positive or negative *Cnr1*$^{loxP/loxP}$ mice. Genotyping for Cre was performed as described previously (1). Since tamoxifen has high binding affinity for CB1R (2) all mice were injected with the same dose of tamoxifen. Generation of conditional beta-cell-specific

knockout (β-CB1R$^{-/-}$) mouse (Cre positive) and its wild-type littermate (β-CB1R$^{+/+}$) mouse (Cre negative) was achieved by i.p. injections with 2 mg/mouse of tamoxifen for 5 days as described previously (1). Excision of *Cnr1* was confirmed by PCR from gDNA extracted from tissue using LOX1/NDL2 primer set (397 bp for positive knockout) and LOX1/SDL2 primer set as loading control (487 bp). Further confirmation was achieved by real time PCR from cDNA using RT-CB1F/RT-CB1R and RT-CreF/RT-CreR primer sets and by immunohistochemistry of the pancreas (Figures S1D-F).

Animal care

All animal procedures and care followed US National Institute of Health guidelines and were approved by the National Institute on Aging Animal Care and Use Committee. Mice were housed in groups of 4 using 12 hrs dark/light cycles, provided with water and fed *ad libitum.* Male mice were fed standard diet (SD; 16.7% kJ fat and 12.4% kJ sugar wt/wt) or high fat-high sugar diet (HFHS; 49.2% kJ fat and 32.2% kJ sugar wt/wt) to induce obesity. Global CB1R knockout (CB1KO) mice backcrossed to a C57Bl/6J background were bred as previously described. At the end of the diet study, *in vivo* tests were performed. Tissues, including pancreas, liver, epididymal and subcutaneous fat, were collected, weighed, and flash frozen or fixed for immunohistochemistry.

Method of randomization

Age and sex matching littermate mice were randomly assigned to vehicle or S961 and to SD or HFHS groups.

Assessment of body composition using nuclear magnetic resonance (NMR) spectroscopy

Mice were weighed and immediately placed on a Bruker mini-NMR (Billerica, MA). Data are presented as % of body weight.

Insulin resistance induction by S961

Miniosmotic pumps (7 days, 1 µl/h) were filled with a total of 10 nmoles of S961. Pumping rate was 0.05 nmoles of S961/h (1.2nmoles/day) (3). Implantation of miniosmotic pumps was performed as previously described (4) and according to Alzet guidelines. Tail vein blood was collected daily until day 6 when mice were injected with 5-Bromo-2′-deoxyuridine (BrdU; 0.1 nmoles/g of body weight) and sacrificed the following day.

Respiratory exchange ratio

Animals were placed in metabolic cages for 48 hrs (Columbus Instruments Comprehensive Lab Monitoring System, Columbus Instruments, Columbus, OH), with *ad lib* access to food and water and controlled light. Metabolic measurements included the respiratory exchange ratio (RER = CO_2 produced / O_2 consumed), activity levels and food and water intake.

Glucose and insulin tolerance tests

Mice were fasted for 4 hrs or overnight and given free access to water, for insulin (ITT) or glucose tolerance tests (IPTT and OGTT) respectively. For ITT responses to pharmacological CB1R blockade, mice were treated with one single dose of JD-5037 [1 mg/kg in a mixture of saline: Tween-80: treatment (94:1:4); (5)] 30 minutes prior to blood draw. Mice were injected i.p. with 1.5 U/kg of insulin and blood glucose was measured at 0, 15, 30, 60 and 90 minutes. For IPGTT and OGTT mice were given i.p. or orally a bolus of 1.5 g/kg glucose and tail vein blood was collected at the timepoints described above. Area under the curve (AUC) was calculated using GraphPad Prism program.

Blood glucose and hormones measurements

Blood glucose from tail vein was measured utilizing an Easygluco blood glucose meter. Blood was collected from tail vein or terminal retro-orbital bleed and heparin or EDTA, aprotinin and DPP4 inhibitors were added. Plasma insulin, GLP-1 and GIP were quantified by ELISA.

Immunohistochemistry of pancreas and islet size quantification

Immunohistochemistry was carried out as previously described (6) with slight modifications. Pancreata were formalin-fixed paraffin-embedded and sectioned at 5 µm. Heat (antigen unmasking solution) or enzymatic-mediated (trypsin 0.05%) antigen retrieval was performed prior to blocking. Primary antibodies were added overnight at 4°C as follows: guinea pig anti-insulin (1:100), mouse anti-glucagon (1:500), rat anti-BrdU (1:250), rabbit anti-CD3 (1:100), mouse anti-CD68 (1:100), rabbit anti-TXNIP (1:250), mouse anti-Ceramide (1:10) and rabbit anti-NF-kB p65 (phospho S536) (1:100). Alexa Fluor (1:1000) or HRP-secondary were incubated 1 hr at room temperature. Nuclei was stained using DAPI for immunofluorescence, and with hematoxylin for DAB staining. DAB staining was performed using DAB Peroxidase Substrate Kit. For anti-mouse secondary antibodies, a mouse-on-mouse (ImmPRES reagent) kit was used to reduce non-specificity. TUNEL staining for apoptosis analysis was carried out using *In Situ* Cell Death

Detection Kit. Imaging was performed at 20x and 40x using a Zeiss LSM-710 confocal microscope (Jena, Germany) and on an inverted Olympus IX51 light microscope. Densitometry of staining was performed using ImageJ (NIH). Islet sizing and beta-cell number were determined using the Pancreas++ (7). The percentage of proliferating beta-cells per islet was determined by counting the number of BrdU-insulin-positive cells and total insulin-positive cells. Ceramide staining was quantified using ImageJ Fiji after transforming original images to 16-bit grayscale.

Immunoblotting

Protein samples extracted from tissues using RIPA buffer containing protease and phosphatase inhibitor cocktails were immunoprecipitated using anti-IRS2 or anti-IR. Samples were then subjected to Tris-glycine PAGE, immunoblotted with anti-IRS2 or anti-p-Tyr and visualized by ECL. Densitometry of bands were quantified using ImageJ.

Islet isolation

Islets were isolated as previously described (8). Mice were sacrificed and pancreas immediately perfused with 0.7 mg/ml collagenase P containing DNAse I in HBSS without calcium and magnesium. Pancreata were incubated 9 min at 37°C with gentle agitation. The enzymatic reaction was quenched with cold HBSS containing 1% (wt/vol) horse serum. Disaggregated pancreata were spun and washed with cold HBSS, and islets were then handpicked. Intra-islet insulin content was extracted by acid ethanol extraction as described previously (9) and static insulin secretion was normalized to content.

Quantification of cAMP in isolated islets

100 islets were lysed with cold 0.1 mol/l HCl and supernatant was used for cAMP detection by Direct cAMP ELISA kit. Response to incretin (exendin 4; Ex-4; 0.33 nM) was performed as previously described (10).

Islet perifusion

Islet perifusion experiments were performed using a mini-perifusion system previously described by us[13]. One hundred islets were placed into insulin secretion assay buffer in polyacrylaminde P4 BioGel fine. The islet-gel mixture was placed into the perifusion column and connected to the perifusion system. Fractions were collected in a 96 well plate every minute for 30 min after switching to the stimulation conditions. Islets were perifused for 1 h in low glucose (2 mmol/l) at a

rate of 100 µl/min, followed by perifusion with stimulatory glucose (7.5 mmol/l; a typical post-prandial glucose level) for 15 min at which point the columns were switched back to basal conditions (2mmol/l glucose). Fractions were collected in a 96 well plate every minute for 30mins from the start of the stimulatory conditions. A dead volume equivalent to 14 mins is noted in the measurement of the glucose concentrations. Insulin concentrations were determined by ELISA.

Oxygen consumption and extracellular acidification rate quantification

Freshly isolated islets (30 islets per well) were plated on an Islet Capture Microplate and measurements of oxygen consumption rate (OCR) and extracellular acidification rate (ECAR) were performed per manufacturer's protocol using a XF^e24 Seahorse Analyzer (Agilent Technologies, Santa Clara, CA).

Mitochondrial reactive oxygen species measurement

Freshly isolated islets from overnight fasted mice were stained for using MitoSOX (5 µmol/l) for 15 min at 37ºC as previously described (11).

Viability assay on isolated islets

Islets were isolated by collagen infusion of pancreas and handpicked. Islets were cultured in Krebs Buffer (130 mmol/l NaCl, 5 mmol/l KCl, 1 mmol/l $H_2NaO_4P\cdot H_2O$, 1 mmol/l $MgSO_4\cdot 7H_2O$, 2 mmol/l $CaCl_2\cdot 2H_2O$, 20 mmol/l HEPES, 25.5 mmol/l $NaHCO_3$, 0.1% (wt/vol) BSA RIA grade) in the presence or absence of 500 µmol/l palmitate and 16.5 mmol/l glucose for 24 hrs (6). Treated isolated islets were plated on a 96-well plate (15 islets per well) and viability was assayed using MultiTox-Fluor Multiplex Cytotoxicity Assay per manufacturer's protocol.

Real Time PCR analysis

Total RNAs were isolated using Trizol or PicoPure RNA Isolation Kit from dissected pancreatic islets. Total RNA concentrations and quality were measured by Nanodrop (Thermo Fisher Scientific). Reverse transcription was performed using SuperScript III First-Strand Synthesis System. Relative expression of selected genes was assayed using TaqMan Fast Advanced Master Mix and FAM-labeled TaqMan Gene Expression Assays on a StepOnePlus Real-Time PCR System (Thermo Fisher Scientific). Duplex reactions were performed using VIC-labeled β-actin for endogenous control.

Phosphoprotein and protein cleavage array

Protein phosphorylation and protein cleavage in isolated islets was performed using PathScan® Array Kit following manufacturer's instructions. Briefly 200 islets were lysed and diluted up to 0.2 mg/ml. Samples were incubated for 2 hrs at room temperature and images were captured using LI-COR Odyssey Sa Infrared Imaging System. Densitometry was analysed using Odyssey Sa Imaging Software.

Cytotoxicity assay on isolated islets

Islets were treated as previously described (12). Briefly, freshly isolated islets were incubated for 18 hours in the absence or prescence of a mixture of cytokines (10 ng/ml IL-1β, 50 ng/ml TNF-α and 50 ng/ml IFN-γ). Cytotoxicity was assayed using MultiTox-Fluor Multiplex Cytotoxicity Assay per manufacturer's protocol.

Microarray analysis

Microarray experiments and analysis were performed and analyzed as previously described (13). Briefly, RNA was extracted from dissected pancreatic islets using Trizol as per the manufacturers recommendations, and RNA concentration and quality evaluated using Nanodrop and the Agilent Bioanalyzer RNA 6000 Chip. Two-hundred ng total RNA was labeled using the Agilent Low-Input QuickAmp Labeling Kit, and was purified and quantified according to the manufacturer's recommendations. A total of 600ng Cy3-labeled cRNA was hybridized for 17hrs to Agilent SurePrint G3 8×60K mouse oligo microarrays (G4852A). Following posthybridization rinses, arrays were scanned using an Agilent SureScan microarray Scanner at 3 micron resolution, and hybridization intensity data extracted from the scanned images using Agilent's Feature Extraction Software. Significant genes were selected by the z-test p value ≤ 0.05, fdr ≤ 0.30 and z-ratio ≥ 1.5. Gene regulatory network and canonic pathway analysis was performed by using Ingenuity Pathway Analysis and gene heatmap by JMP program. The data has been deposited at GEO (GSE102027).

Endocannabinoid (EC) levels in plasma

20μl of plasma was added to 500 μl of cold methanol/Tris buffer [50mmol/l, pH8] containing internal standards. Subsequently to each mixture, 1.5 ml ice-cold methanol–chloroform mixture

(2:1) with 0.5 ml of Tris buffer [50mmol/l, pH8] was added. The mixture was centrifuged at 500g for 2 minutes, and the chloroform phase was removed to a glass tube. The extraction was repeated twice. The combined extract was dried and reconstituted in chloroform. Acetone was added to all the samples and the solution was centrifuged at 16,000 x g for 5 minutes. The supernatant was collected, dried and reconstituted in methanol. ECs were analyzed by LC-MS/MS. The mobile phase consisted of water with 0.1% formic acid as component A and acetonitrile with formic acid as component B. A linear gradient was run as follows: 0 min 60% B; 1 min 60% B; 7 min 90% B;.10 min 90% B; 10.1 min 90% B; 14.9 min 60% B at a flow rate of 0.8 ml/min with a total run time of 15 min. MS/MS analysis was performed using API 5500 QTRAP. The data was acquired and analyzed using Analyst version 1.5.1. Positive electrospray ionization data were acquired using multiple reactions monitoring (MRM). The TIS instrumental source settings for temperature, curtain gas, ion source gas 1 (nebulizer), ion source gas 2 (turbo ion spray) and ion spray voltage were 550˚C, 30 psi, 60 psi, 60 psi and 5500 V, respectively. The standards were characterized using the following MRM ion transitions and TIS compound parameters (declustering potential, collision energy and cell exit potential): AEA: 348.2/62.1 (80V, 18V, 10V); AEA d4: 353.1/66.1 (80V, 18V, 10V); 2AG: 379.4/287.2 (80V, 20V,10V); 2AGd5: 384.4/287.4 (80V, 20.V, 10V).

Flow Cytometry

Pancreatic lymphocytes were isolated after pancreas perfusion as described above for islet isolation. Cells were washed 3 times with media (RPMI) by centrifuging 5 min at 1500 x rpm. Then, erythrocytes were lysed by incubating cells incubated with ACK lysis buffer for 3 min at 24ºC. After washing 3 times with media, cells were plated at a density of $2x10^5$ cells per well in a 96-well plate, washed 2 times with FACS buffer containing PBS, 0.05% BSA and 0.5% and NaN_3, and blocked for 5 min with 0.1% goat serum. The staining was carried out by using PE-anti mouse CD8a and FITC anti-mouse CD69 that were incubated for 20 min at 24ºC. Appropriate isotype controls were used in all the experiments. Populations were determined by using BD FACS Canto II and the software FACS Diva.

ESM Table 1. Antibodies and dilutions

Antibodies and dilution	SOURCE	CATALOG NUMBER
Guinea pig anti-insulin (1:100)	Dako (Carpinteria, CA)	A0564
Mouse anti-glucagon (1:500)	Sigma-Aldrich (St Louis, MI)	SAB4501137
Rat anti-BrdU (1:100)	Accurate Chemicals (Westbury, NY)	H9970
Rabbit anti-CD3 (1:100)	Abcam (Cambridge, MA)	Ab5690
Mouse anti-CD68 (1:100)	Abcam	Ab31630
Rabbit anti-TXNIP (1:100)	Abcam	Ab188865
Mouse anti-Ceramide (1:10)	Enzo Life Sciences (Farmingdale, NY)	ALX-804-196-T050
Rabbit anti-NF-kB p65 (phospho S536) (1:100)	Abcam	Ab86299
Alexa Fluor secondary antibodies (1:1000)	Thermo Fisher Scientific (Waltham, MA)	A21450/A11029/A11036/ A21245/A11073/A21236/ A11077/A11057
HRP-secondary (1:5000)	Thermo Fisher Scientific	NA931V/NA934V
Rabbit anti-IRS2 (1:1000)	Cell Signaling (Danvers, MA)	3089
Insulin receptor beta antibody (1 μg)	Santa Cruz Biotechnology (Dallas, TX)	SC-711
p-Tyr (PY20) (1:1000)	Santa Cruz Biotechnology	SC-508
PE-anti mouse CD8a	Biolegend (San Diego, CA)	clone 53-6.7
FITC anti-mouse CD69	Biolegend	clone H1.2F3

ESM Table 2. Chemicals, peptides, and recombinant Proteins

Name	SOURCE	CATALOG NUMBER
Insulin receptor antagonist S961	Novo Nordisk (14)	S961
Exendin-4	Sigma-Aldrich	E7144
Tamoxifen	Sigma-Aldrich	T5648
Palmitate	Sigma-Aldrich	P0500
5-Bromo-2′-deoxyuridine (BrdU)	Thermo Fisher Scientific	00-0103
Insulin	Novo Nordisk (Plainsboro, NJ)	Novolin
Collagenase P	Roche	11213873001
DNAse I	Sigma Aldrich	D4527-200KU
HBSS	Thermo Fisher Scientific	14025-076
Antigen unmasking solution	Vector Labs	H-3300
DAPI	Thermo Fisher Scientific	D1306
DAB Peroxidase substrate kit	Vector Labs	SK-4100
RIPA buffer	Boston BioProducts	BP-115DG
Protease inhibitor cocktails	Sigma-Aldrich	P8340
Phosphatase inhibitor cocktails II and III	Sigma-Aldrich	P5726/P0044
MitoSOX	Thermo Fisher Scientific	M36008
TRIzol reagent	Thermo Fisher Scientific	15596018
JD-5037	Jenrin Discovery (15) (Wilmington, PA)	
IL-1β	Sigma-Aldrich	I5271
TNF-α	Sigma-Aldrich	T7539

IFN-γ	Sigma-Aldrich	I4777

ESM Table 3. Critical commercial assays

Name	SOURCE	CATALOG NUMBER
Ultrasensitive mouse insulin ELISA	Crystalchem	90080
Active GLP-1 ELISA	Alpco	43-GP1HU-E01
GIP ELISA	Millipore	MGTMAG-78K
In Situ Cell Death Detection Kit	Roche	12 156 792 910
ImmPRES Reagent kit	Vector Labs	MP-7402
PicoPure RNA Isolation Kit	Thermo Fisher Scientific	KIT0214
SuperScript III	Thermo Fisher Scientific	18080-051
TaqMan Fast Advanced Master Mix	Thermo Fisher Scientific	4444963
TaqMan Gene Expression Assays	Thermo Fisher Scientific	4331182
MultiTox-Fluor Multiplex Cytotoxicity Assay	Promega	G9201
Direct cAMP ELISA	Enzo Life Sciences	ADI-900-066
Low-Input QuickAmp Labeling Kit	Agilent Technologies	5190
SurePrint G3 8×60K mouse oligo microarrays	Agilent Technologies	G4852A
PathScan® Array Kit (Fluorescent Readout)	Cell Signaling	7744

ESM Table 4. Experimental models

Strains (Organisms; sex)	SOURCE
Cnr1$^{flox/wt}$ (mice)	Gonzalez-Mariscal I. Baltimore, MD, USA
MIP-Cre/ERT (mice; males)	(1), University of Chicago, Chicago, IL, USA
β-CB1R$^{+/+}$ (mice; males)	Gonzalez-Mariscal I. Baltimore, MD, USA
β-CB1R$^{-/-}$ (mice; males)	Gonzalez-Mariscal I. Baltimore, MD, USA
CB1KO (mice; males)	(16), NIH, Bethesda, MD, USA

ESM Table 5. Oligonucleotides

Name	SOURCE
LOX1 CTGGACAGCTCATCCTTGGTTCTATAC	In this study
NDL2 CAAGAGACTGCAGCAGGCCAAATCTAG	In this study
SDL2 TGAGTGGCAAGAAAAGTCAGAGTG	In this study
RT-CB1F AAGTCGATCTTAGACGGCCTT	In this study
RT-CB1R TCCTAATTTGGATGCCATGTCTC	In this study
RT-CreF AGGTTCGTTCACTCATGGA	In this study
RT-CreR TCGACCAGTTTAGTTACCC	In this study

ESM Table 6. Softwares

Name	SOURCE
GraphPad Prism 6.07	GraphPad Software Inc. (La Jolla, CA)
Pancreas++	NIH (7) (Baltimore, MD)

ImageJ 1.48v	NIH; https://imagej.nih.gov
Analyst version 1.5.1	Applied Biosystems (Thermo Fisher Scientifics)
Ingenuity Pathway Analysis	Qiagen (Germantown, MD
JMP 6.0	SAS Institute, Inc
FACS Diva version 6.1.3.	BD Bioscience (Franklin Lakes, NJ)

ESM Table 7. Other materials

Name	SOURCE	CATALOG NUMBER
Miniosmotic pumps	Alzet (Cupertino, CA)	Model 2001
Easygluco blood glucose meter	US Diagnostics	
Tris-glycine PAGE	Thermo Fisher Scientific	EC60385BOX
ECL	GE Healthcare	RPN2209
Islet Capture Microplate	Agilent	101122-100
Standard diet	Dyets Inc.	101845
High Fat High Sugar diet	Dyets Inc.	103806
Polyacrylaminde P4 BioGel fine	Biorad	
Perifusion column	Biorep Technologies	
FACS Canto II	BD Bioscience	

1. Wicksteed B et al. Conditional gene targeting in mouse pancreatic ß-Cells: analysis of ectopic Cre transgene expression in the brain.. *Diabetes* 2010;59(12):3090–8.

2. Ford BM, Franks LN, Radominska-Pandya A, Prather PL. Tamoxifen Isomers and Metabolites Exhibit Distinct Affinity and Activity at Cannabinoid Receptors: Potential Scaffold for Drug Development. *PLoS One* 2016;11(12):e0167240.

3. Yi P, Park J-S, Melton DA. Betatrophin: a hormone that controls pancreatic β cell proliferation.. *Cell* 2013;153(4):747–58.

4. Wang Y et al. Glucagon-like peptide-1 can reverse the age-related decline in glucose tolerance in rats.. *J. Clin. Invest.* 1997;99(12):2883–9.

5. Dossou KSS et al. Development and preliminary validation of a plate-based CB1/CB2 receptor functional assay. *Anal Biochem* 2013;437(2):138–143.

6. Fiori JL et al. Resveratrol prevents β-cell dedifferentiation in nonhuman primates given a high-fat/high-sugar diet.. *Diabetes* 2013;62(10):3500–13.

7. Chen H et al. Pancreas++: Automated Quantification of Pancreatic Islet Cells in Microscopy Images. *Front. Physiol.* 2013;3(482):3.

8. Li D-S, Yuan Y-H, Tu H-J, Liang Q-L, Dai L-J. A protocol for islet isolation from mouse pancreas. *Nat. Protoc.* 2009;4(11):1649–1652.

9. Carlsson A, Hallgren I-B, Johansson H, Sandler S. Concomitant Enzyme-Linked Immunosorbent Assay Measurements of Rat Insulin, Rat C-Peptide, and Rat Proinsulin from Rat Pancreatic Islets: Effects of Prolonged Exposure to Different Glucose Concentrations. *Endocrinology* 2010;151(10):5048–5052.

10. González-Mariscal I et al. Human CB1 Receptor Isoforms, present in Hepatocytes and β-cells, are Involved in Regulating Metabolism.. *Sci. Rep.* 2016;6:33302.

11. Tang C et al. Evidence for a Role of Superoxide Generation in Glucose-Induced -Cell Dysfunction In Vivo. *Diabetes* 2007;56(11):2722–2731.

12. Li L, El-Kholy W, Rhodes CJ, Brubaker PL. Glucagon-like peptide-1 protects beta cells from cytokine-induced apoptosis and necrosis: role of protein kinase B. *Diabetologia* 2005;48(7):1339–1349.

13. Cheadle C, Vawter MP, Freed WJ, Becker KG. Analysis of Microarray Data Using Z Score Transformation. *J. Mol. Diagnostics* 2003;5(2):73–81.

14. Vikram A, Jena G. S961, an insulin receptor antagonist causes hyperinsulinemia, insulin-resistance and depletion of energy stores in rats.. *Biochem. Biophys. Res. Commun.* 2010;398(2):260–5.

15. Chorvat RJ, Berbaum J, Seriacki K, McElroy JF. JD-5006 and JD-5037: Peripherally restricted (PR) cannabinoid-1 receptor blockers related to SLV-319 (Ibipinabant) as metabolic disorder therapeutics devoid of CNS liabilities.. *Bioorg. Med. Chem. Lett.* 2012;22(19):6173–80.

16. Zimmer A, Zimmer AM, Hohmann AG, Herkenham M, Bonner TI. Increased mortality, hypoactivity, and hypoalgesia in cannabinoid CB1 receptor knockout mice. *Proc. Natl. Acad. Sci.* 1999;96(10):5780–5785.

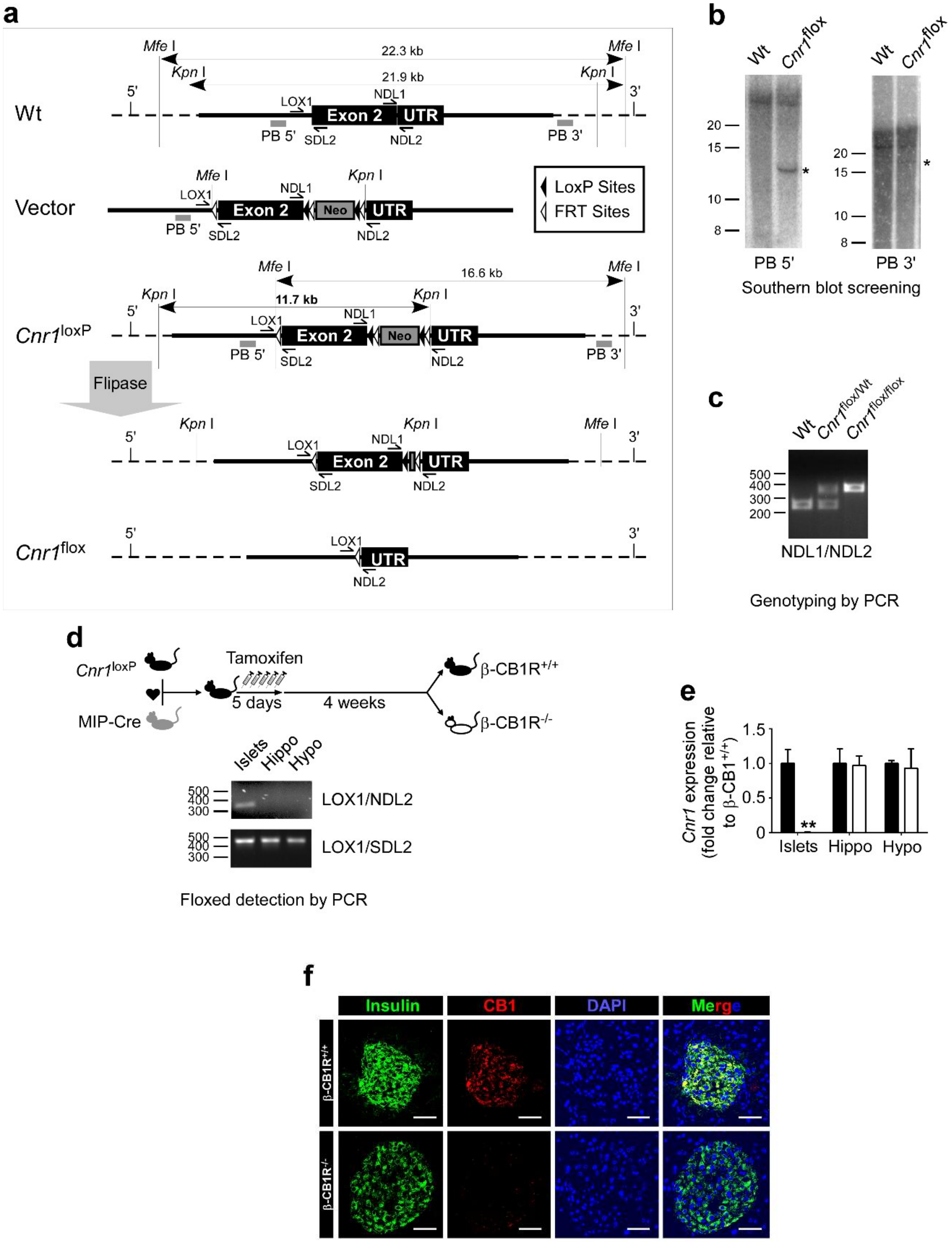
a
Wt
Vector
Cnr1loxP
Flipase
Cnr1flox
Mfe I
Kpn I
22.3 kb
21.9 kb
16.6 kb
11.7 kb
5'
3'
LOX1
NDL1
SDL2
NDL2
Exon 2
UTR
Neo
PB 5'
PB 3'
LoxP Sites
FRT Sites
b
Wt
Cnr1flox
PB 5'
PB 3'
Southern blot screening
c
Cnr1flox/Wt
Cnr1flox/flox
NDL1/NDL2
Genotyping by PCR
d
MIP-Cre
Tamoxifen
5 days
4 weeks
β-CB1R+/+
β-CB1R-/-
Islets
Hippo
Hypo
LOX1/NDL2
LOX1/SDL2
Floxed detection by PCR
e
Cnr1 expression (fold change relative to β-CB1+/+)
f
Insulin
CB1
DAPI
Merge

ESM Fig. 1

ESM Fig. 1. Generation of Cre-conditional *Cnr1*-floxed mouse strain. (a) Schematic of wild type *Cnr1* gene (Wt), targeting vector (Vector), *Cnr1* allele flanked by LoxP sites ($Cnr1^{LoxP}$) and null *Cnr1* allele ($Cnr1^{flox}$). Black solid line, homology arms; grey bars, Southern blot probes (PB); black triangles, LoxP sites; white triangles, FRT sites; grey box, Neomycin cassette (Neo); half arrow, PCR primers; normal arrows, length of digested products. (b) Southern blot screening of 5' and 3' recombination using Kpn I and Mfe I restriction enzymes with expected sizes of 11.7 kb and 16.6 kb respectively. (c) PCR of wild type, heterozygous and homozygous for the floxed-*Cnr1* allele. Expected sizes are 367 bp for wild type and 255 bp for the floxed allele. (d) PCR analysis of β-$CB1R^{-/-}$ with LOX1/NDL2 and LOX1/SDL2 primers (397 bp if floxed) and LOX1/NDL2 primers (488 bp) in islets, hippocampus (Hippo) and hypothalamus (Hypo) gDNA. (e) Expression of *Cnr1* mRNA in isolated islets compared to different brain areas in β-$CB1R^{+/+}$ (black bars) and β-$CB1R^{-/-}$ mice (white bars) one month after tamoxifen injection. (F) Immunostaining for insulin (green), CB1R (red) and DAPI (blue) and merged images of pancreas from β-$CB1R^{+/+}$ and β-$CB1R^{-/-}$ mice one month after tamoxifen injections (scale bar = 50μm). N = 6-7 mice. Data show mean ± SEM. Significance by t-test *p ≤ 0.05, **p ≤ 0.01 vs β-$CB1R^{+/+}$.

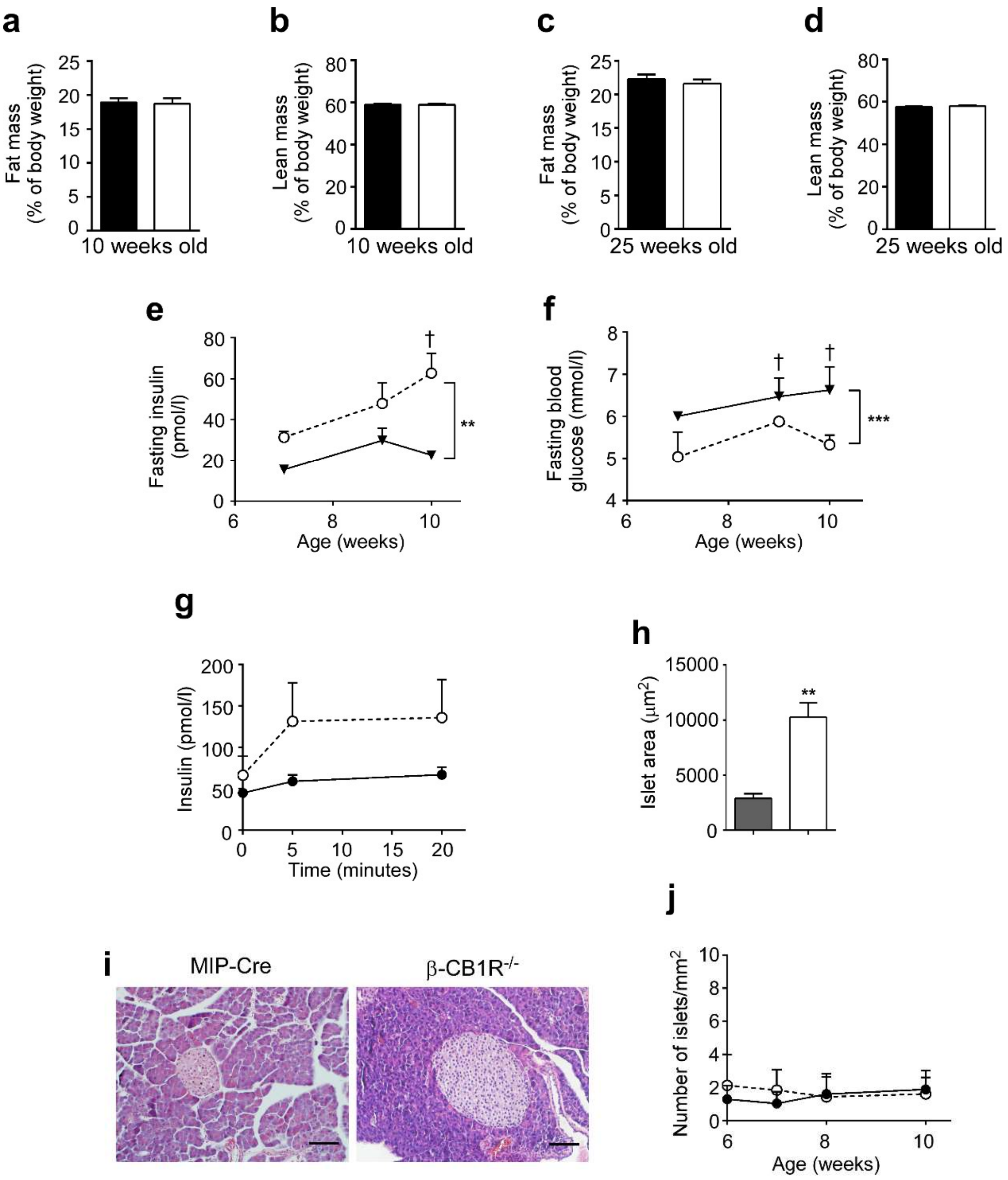
a
Fat mass
(% of body weight)
10 weeks old
b
Lean mass
(% of body weight)
10 weeks old
c
Fat mass
(% of body weight)
25 weeks old
d
Lean mass
(% of body weight)
25 weeks old
e
Fasting insulin
(pmol/l)
Age (weeks)
f
Fasting blood
glucose (mmol/l)
Age (weeks)
g
Insulin (pmol/l)
Time (minutes)
h
Islet area (μm2)
i
MIP-Cre
β-CB1R-/-
j
Number of islets/mm2
Age (weeks)

ESM Fig. 2

ESM Fig. 2. Phenotype of β-CB1R$^{-/-}$ and MIP-Cre/ERT mice. (a-d) β-CB1R$^{+/+}$ (black bars; black circles, solid line) and β-CB1R$^{-/-}$ (white bars; white circles, dashed line) mice were injected with tamoxifen at 6 weeks of age and (a,c) fat and (b,d) lean mass measured by NMR at (a,b) 10wks and (c,d) 25wks of age. (e) Fasting plasma insulin and (f) blood glucose in MIP-Cre/ERT (MIP-Cre; black triangles, solid line) and β-CB1R$^{-/-}$ mice (white circles, dashed line) at times indicated after tamoxifen injection. (g) Mixed glucose-lipid stimulated insulin secretion were examined at 25wks of age in β-CB1R$^{+/+}$ (black circles, solid line) and β-CB1R$^{-/-}$ (white circles, dashed line) mice. (h) Islet area and (i) hematoxylin-eosin staining of pancreas of 12-15-week old MIP-Cre (grey bars) and β-CB1R$^{-/-}$ (white bars) mice. (j) Quantification of islet density in pancreas from β-CB1R$^{+/+}$ (black circles, solid line) and β-CB1R$^{-/-}$ (white circles, dashed line) mice at times indicated after tamoxifen injection. N = 6-7 mice. Data show mean ± SEM. Significance by ANOVA or t-test *p ≤ 0.05, **p ≤ 0.01 compared to β-CB1R$^{+/+}$ and †p ≤ 0.05 compared to week 0. Scale bar = 50 µm. Data shown mean ± SEM (n=4-6 mice/treatment). Significance by ANOVA **p≤0.01, ***p≤<0.001 vs β-CB1R$^{+/+}$; †p≤0.05 vs week 0.

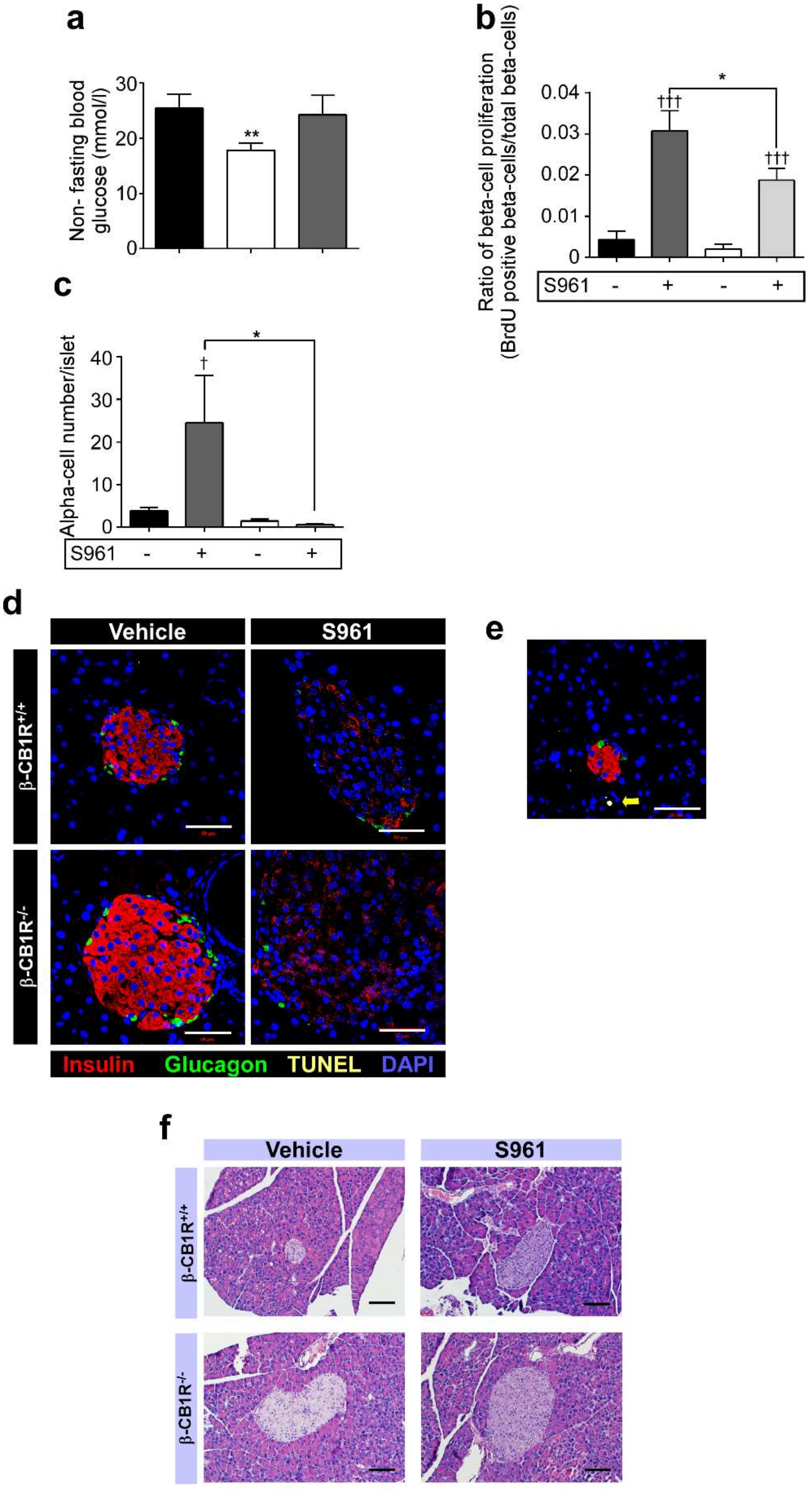
a
Non- fasting blood glucose (mmol/l)
30
20
10
0
**
b
Ratio of beta-cell proliferation (BrdU positive beta-cells/total beta-cells)
0.04
0.03
0.02
0.01
0
*
†††
†††
S961 - + - +
c
Alpha-cell number/islet
40
30
20
10
0
†
*
S961 - + - +
d
Vehicle
S961
β-CB1R+/+
β-CB1R-/-
Insulin Glucagon TUNEL DAPI
e
f
Vehicle
S961
β-CB1R+/+
β-CB1R-/-

ESM Fig. 3

ESM Fig. 3. S961 does not induce apoptosis or immune cell infiltration in pancreas. Miniosmotic pumps containing vehicle or 10 nmoles of S961 (1.2 nmoles/day) were implanted subcutaneously in 10-12 week old β-CB1R$^{+/+}$ (black bars), β-CB1R$^{-/-}$ (white bars) and MIP-Cre/ERT (dark grey bars) mice one month after tamoxifen injections. (a) Non-fasting blood glucose at the end of the study. (b) Quantification of alpha-cell number and per islet and (c) beta-cell proliferation (BrdU positive beta-cells per total number of beta-cells) in β-CB1R$^{+/+}$ (vehicle = black circles, solid line and black bars; S961 = black triangles, solid line and dark grey bars) and β-CB1R$^{-/-}$ (vehicle = white circles, dashed line and white bars; S961 = white triangles, dashed line and light grey bars) mice treated with S961. (d,e) Immunostaining for TUNEL (white; yellow arrow), insulin (red), glucagon (green), and DAPI (blue) of pancreas from β-CB1R$^{+/+}$ and β-CB1R$^{-/-}$ mice treated with vehicle or S961 for 6 days (scale bar = 50μm). Panel (e) shows positive TUNEL staining in a non-islet cell. (f) Hematoxylin-eosin staining of pancreas from β-CB1R$^{+/+}$ and β-CB1R$^{-/-}$ mice treated with vehicle or S961 for 6 days (scale bar = 100 μm). Data shown mean ± SEM. Significance by ANOVA, *p≤0.05, **p≤0.01 vs β-CB1R$^{+/+}$ and †p≤0.05, †††p≤0.001 vs vehicle.

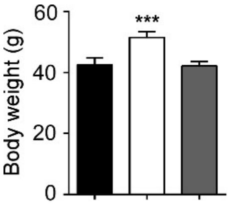

ESM Fig. 4

ESM Fig. 4. β-CB1R$^{-/-}$ mice become more obese than MIP-Cre/ERT and β-CB1R$^{+/+}$ mice when placed on a high fat high sugar diet. 6-8-week old mice were injected with tamoxifen and one month later were placed on a high fat high sugar diet (HFHS) for 15 weeks. (a) Body weight is shown for β-CB1R$^{+/+}$ (black bars), β-CB1R$^{-/-}$ (white bars) and MIP-Cre/ERT (dark grey bars). N = 7-9 mice/group. Data shown mean ± SEM. Significance by ANOVA, ***p<0.001.

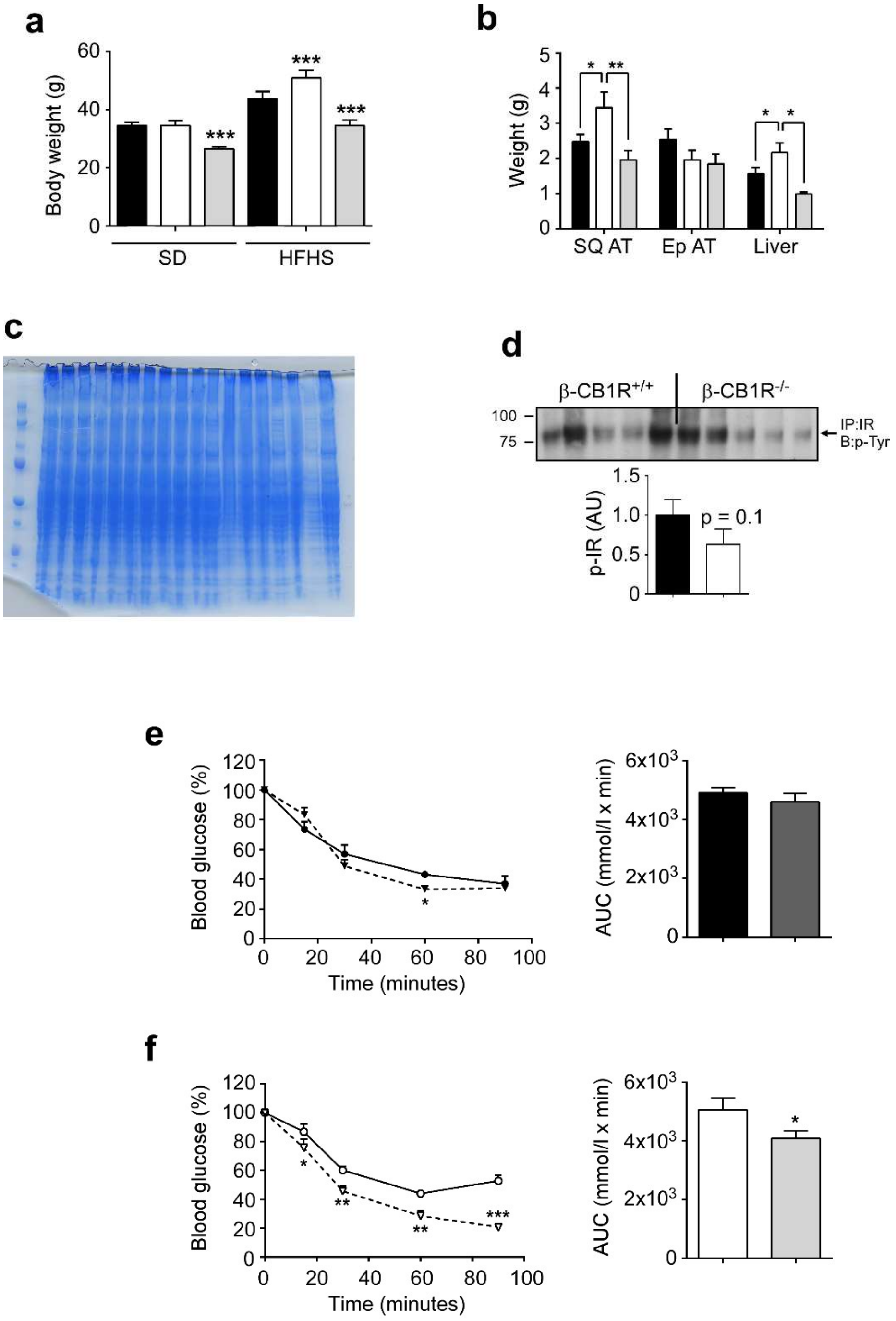

a
Body weight (g)
SD
HFHS
***
b
Weight (g)
SQ AT
Ep AT
Liver
c
d
β-CB1R+/+
β-CB1R-/-
100
75
IP:IR
B:p-Tyr
p-IR (AU)
p = 0.1
e
Blood glucose (%)
Time (minutes)
AUC (mmol/l x min)
f
Blood glucose (%)
Time (minutes)
AUC (mmol/l x min)

ESM Fig. 5

ESM Fig. 5. Obesity and insulin resistance in β-CB1R$^{-/-}$ mice fed a HFHS. (a) Body weight and (b) liver, epididymal (Ep) and subcutaneous (SQ) adipose tissue (AT) weight of β-CB1R$^{+/+}$ (black bars), β-CB1R$^{-/-}$ (white bars) and CB1KO (light grey bars) mice fed a HFHS for 15 weeks. (c) Coomassie blue staining of the liver extracts from β-CB1R$^{+/+}$ and β-CB1R$^{-/-}$ HFHS and SD mice that were used for immunoprecipitating IRS-2 (Figure 4D). (d) Western blot analysis of p-IR immunoprecipitated from liver extracts (top panel) and band density analysed by Image-J (bottom panel) from HFHS-fed β-CB1R$^{+/+}$ (black bars) and β-CB1R$^{-/-}$ mice (white bars). (e, f) Mice fasted for 4hrs were injected with JD-5037 for 30min prior an insulin tolerance test (ITT) and blood glucose was measured. (e) ITT in HFHS-β-CB1R$^{+/+}$ (JD-5037 = dashed line; grey bar) and (f) in HFHS-β-CB1R$^{-/-}$ (JD-5037 = dashed line, grey bar). N = 7 mice. Data show mean ± SEM. Significance by ANOVA or t-test, *$p \leq 0.05$, **$p \leq 0.01$, ***$p \leq 0.001$.

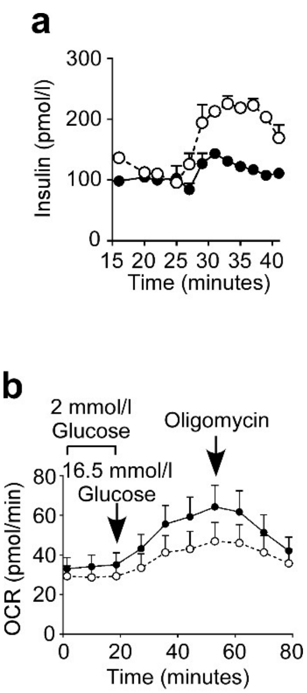

ESM Fig. 6

ESM Fig. 6. β-CB1R$^{-/-}$ islets have a metabolic shift. (a) Glucose-stimulated insulin secretion from perifused wild type (black circles, solid line) and CB1KO (white circles, dashed line) islets (N = 100 islets; N = 4 mice). (b) Freshly isolated islets (n = 30) were incubated in 2mmol/l glucose and oxygen consumption rate (OCR) was measured when stimulated with 16.5mmol/l glucose by SeaHorse technology. N= 30-100 islets, N = 6-8 replicates. Data show mean±SEM.

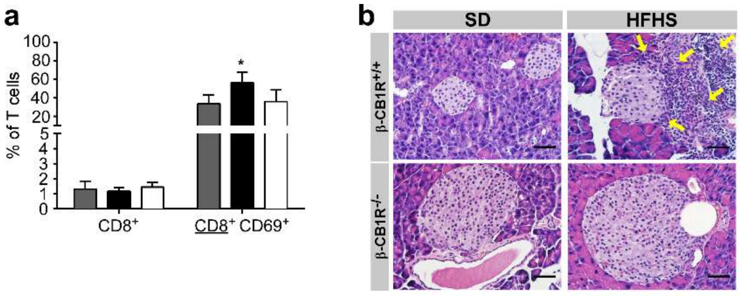

e

Islets
β-CB1R$^{+/+}$ - β-CB1R$^{-/-}$

Pathways	z score	p-value
CB1	-1.25	$6 \cdot 10^{-3}$
TXNIP	-1.60	$9 \cdot 10^{-3}$
NF-κB	-2.46	$4 \cdot 10^{-6}$
TNF	-1.60	$6 \cdot 10^{-6}$
IL-1β	-1.68	$4 \cdot 10^{-9}$

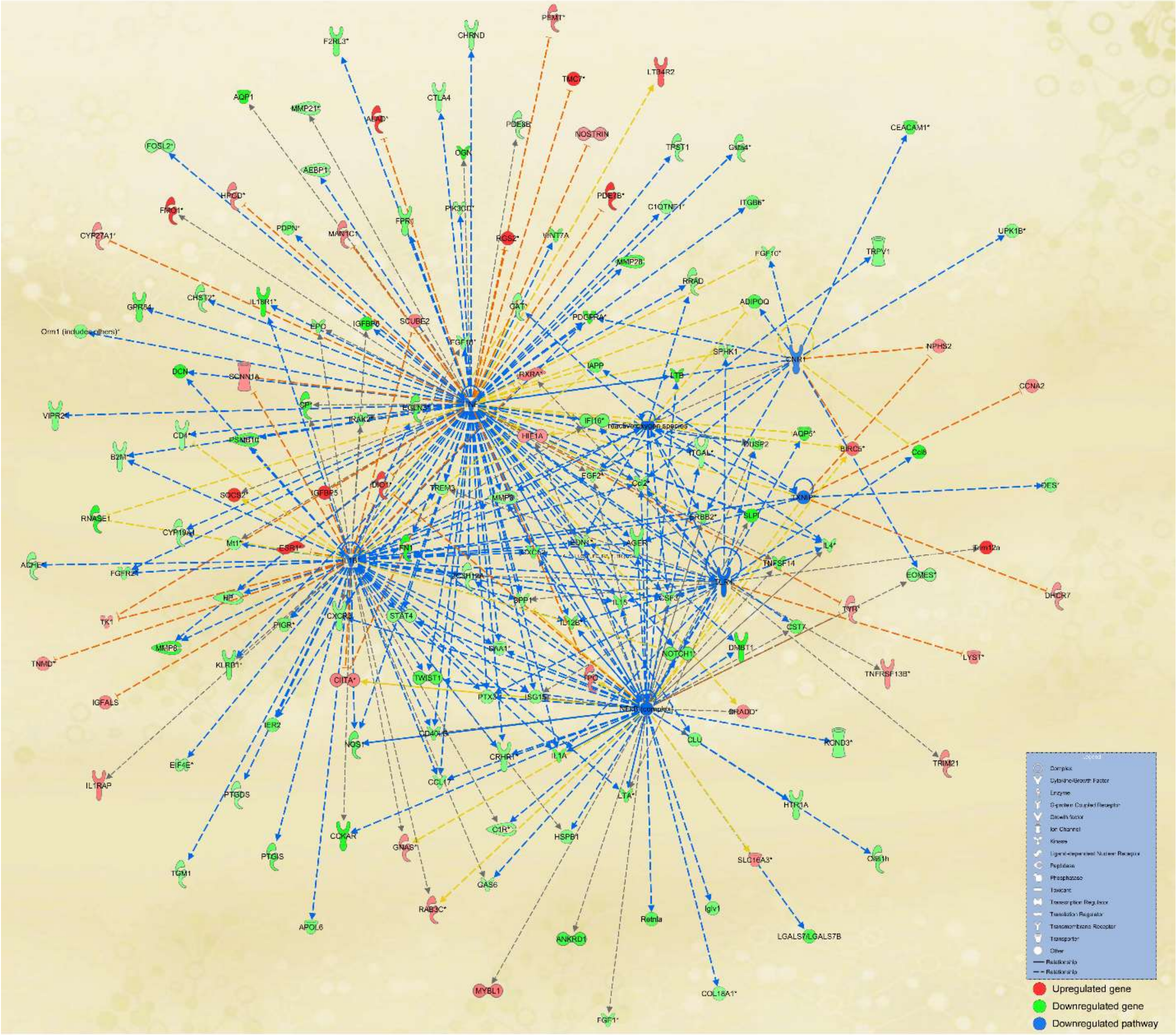

ESM Fig. 7

ESM Fig. 7. β-CB1R$^{-/-}$ mice are protected from diet-induced insulitis. (a) Flow cytometry analysis of pancreatic $CD8^+$ and $CD8^+$-$CD69^+$ cells isolated from SD-fed β-CB1R$^{+/+}$ (dark grey bars), HFHS-fed β-CB1R$^{+/+}$ (black bars) and HFHS-fed β-CB1R$^{-/-}$ mice (black bars) (N = 3-6 mice). (b) Hematoxylin and eosin staining of pancreas from β-CB1R$^{+/+}$ and β-CB1R$^{-/-}$ mice fed a SD or HFHS for 15 weeks (scale bar = 50 µm). Yellow arrows show infiltrated immune cells around the islet. (c) Circulating endocannabinoid levels in plasma from β-CB1R$^{+/+}$ (black bars) and β-CB1R$^{-/-}$ (white bars) mice fed a HFHS for 15 weeks. (d) Relative *Cnr2* expression in freshly isolated β-CB1R$^{+/+}$ (black bar) and β-CB1R$^{-/-}$ (white bar) islets. (e) Microarray gene analysis was carried out on total RNA extracted from freshly isolated islets of β-CB1R$^{+/+}$ and β-CB1R$^{-/-}$ mice: selected pathways related to inflammation and gene expression profile (red, significantly upregulated; blue, significantly downregulated). N = 6-7 mice per group. Data are mean ± SEM. Significance by ANOVA or t-test, $*p \leq 0.05$.
